# Gene–Physical Activity Interplay in Depression: Candidate–Gene Interactions, Polygenic Susceptibility, Lifestyle Context, and Mendelian Randomization Evidence—Systematic Review

**DOI:** 10.3390/jcm15135025

**Published:** 2026-06-27

**Authors:** James Chmiel, Marta Kopańska

**Affiliations:** 1 Faculty of Physical Culture and Health, Institute of Physical Culture Sciences, University of Szczecin, 71-065 Szczecin, Poland; 2 Department of Medical Communication and Professional Competency Development, Faculty of Medicine, Collegium Medicum, University of Rzeszów, 35-310 Rzeszów, Poland; mkopanska@ur.edu.pl

**Keywords:** depression, physical activity, genetic, genes, candidate genes, polygenic susceptibility, mendelian randomization, lifestyle

## Abstract

**Background/Objectives**: Depression is a heterogeneous disorder shaped by both inherited liability and environmental exposures. Physical activity is a scalable, modifiable behavior consistently linked to lower depressive symptoms and reduced incident depression, but interpretation is complicated by measurement error, genetic confounding, and bidirectional pathways in which depression can also reduce activity. This systematic review synthesizes evidence on gene–physical activity interplay in depression across four complementary frameworks: (i) candidate–gene interaction studies, (ii) genome-wide susceptibility indexed by depression polygenic risk scores (PRS), (iii) lifestyle-context and activity-architecture analyses (e.g., timing and accumulation patterns), and (iv) Mendelian randomization (MR) studies testing bidirectional causal effects between activity-related traits and depression. **Methods**: A PRISMA-aligned search and narrative synthesis were conducted due to substantial heterogeneity in populations, exposure measurement (self-report vs. accelerometer), genetic approaches, and depression phenotypes. Twenty-seven studies met inclusion criteria. **Results**: Across designs, the most consistent pattern was that higher physical activity (or lower inactivity) tracked with lower depression risk or symptom burden even when genome-wide genetic susceptibility was modeled, supporting largely additive contributions of PRS and activity rather than strong, generalizable PRS × activity interactions. MR evidence most consistently supported a protective effect of physical activity on depression when activity was indexed by accelerometer-derived phenotypes, whereas self-reported activity instruments yielded weaker or more heterogeneous findings. Bidirectional genetic evidence also indicated that depression liability can causally suppress physical activity, consistent with a feedback loop relevant for prevention and intervention. Candidate-gene moderation effects were mixed and typically emerged only in specific contexts (e.g., stress history, developmental stage, sex, or treatment setting), underscoring limited replicability and sensitivity to how activity is operationalized. **Conclusions**: Overall, the literature supports physical activity as broadly protective across levels of genetic risk, while emphasizing that robust inference depends on objective exposure measurement, careful handling of confounding and reverse causation, and improved generalizability beyond predominantly European-ancestry genetic resources.

## 1. Introduction

Depression is a common and disabling psychiatric disorder marked by substantial heterogeneity in symptom profiles, course, and treatment response. Etiologically, depression reflects the joint influence of inherited liability and environmental exposures operating across the life span. Meta-analytic evidence from family and twin studies indicates that major depression is moderately heritable, while a large proportion of variance is attributable to nonshared environmental influences [1]. Consistent with this, contemporary genomic research shows that depression has a highly polygenic architecture, with risk distributed across many common variants of small effect rather than explained by a small number of major loci [2,3].

Physical activity is a central exposure in this context because it is modifiable, scalable, and measurable across multiple domains (structured exercise, habitual activity, cardiorespiratory fitness, and sedentary behavior). Across prevention and treatment literatures, higher physical activity and exercise interventions are generally associated with lower depressive symptom burden and reduced risk of incident depression, although effect sizes vary and between-person heterogeneity is expected [4,5]. Importantly, activity–depression associations are methodologically challenging. Physical activity is socially patterned and correlated with health status and functional capacity, and depression itself can reduce motivation and energy, complicating temporal ordering and causal interpretation. Measurement quality further contributes to heterogeneity: compared with direct (device-based) assessment, self-reported activity exhibits substantial error and can differ systematically from objective estimates, which tends to attenuate associations and makes effect modification analyses especially difficult [6].

A mechanistic account of why physical activity relates to depression plausibly involves multiple partially independent biological and psychosocial pathways that may differ in salience across individuals and clinical presentations. Proposed biological channels include neuroplasticity-related processes (often framed within neurotrophic models of stress-related mood disorders) [7], immune–inflammatory signaling implicated in a subset of depressive phenotypes [8], and skeletal-muscle-mediated metabolic routes, including exercise-induced shifts in kynurenine metabolism that have been linked to stress resilience in experimental work [9]. Physical activity can also influence affect and stress responsivity via neuromodulatory systems; for example, evidence supports a role of endocannabinoid signaling in exercise-related anxiolysis and analgesia in animal models, with convergent human data indicating exercise-associated endocannabinoid changes [10,11]. In addition, activity can shape sleep and circadian regulation—systems that are tightly coupled to mood—because exercise can act as a zeitgeber capable of shifting circadian phase depending on timing and internal biological time [12,13]. Psychosocial mechanisms, including behavioral activation, self-efficacy, social engagement, and reductions in rumination, may operate alongside these biological pathways and help explain why benefits can emerge even at modest activity doses and across diverse activity types [14].

Interpreting depression–activity evidence also requires recognizing that physical activity is not a purely environmental exposure: behavioral genetic work indicates a meaningful genetic contribution to interindividual differences in physical activity across the life span, implying potential for gene–environment correlation and familial confounding in observational designs [15,16]. Consequently, research that integrates genetic liability with physical activity must be interpreted in light of (i) depression’s polygenic architecture, (ii) heterogeneity in depression phenotypes and activity operationalizations, (iii) measurement error—especially in self-report exposures—and (iv) bidirectional and confounded pathways linking mood and behavior.

Given this rapidly evolving and methodologically diverse evidence base, an integrated synthesis is needed. The present systematic review therefore evaluates evidence on gene–physical activity interplay in depression across four complementary frameworks: (i) candidate–gene interaction studies, (ii) genome-wide liability approaches using depression PRS, (iii) lifestyle-context and activity-architecture analyses (including timing/patterning), and (iv) MR studies testing bidirectional causal effects between activity-related traits and depression. By triangulating across these designs—each with distinct strengths and vulnerabilities—this review aims to clarify which findings appear most consistent and robust, under what contexts moderation signals emerge, and what the combined literature implies about genetic susceptibility, modifiable behavior, and causal directionality in depression.

## 2. Materials and Methods

### 2.1. Review Design and Reporting

This systematic review synthesizes evidence on the interplay between genetic factors and physical activity-related exposures in relation to depression. The review was planned and reported in line with PRISMA guidance for systematic reviews, with particular attention to genetic epidemiology and causal-inference approaches (polygenic risk scoring and Mendelian randomization). The PRISMA checklist is presented in Supplementary Table S1.

### 2.2. Literature Search Strategy

A comprehensive search strategy was developed to capture three overlapping evidence streams: (i) candidate–gene and gene × physical activity interaction studies, (ii) studies incorporating genome-wide genetic liability (e.g., depression polygenic risk scores) alongside physical activity or lifestyle measures, and (iii) Mendelian randomization studies evaluating bidirectional effects between physical activity (or inactivity/sedentary behavior) and depression. The following electronic databases were searched from inception to January 2026: MEDLINE/PubMed, Embase, PsycINFO, Scopus/Web of Science Core Collection, and Cochrane CENTRAL. No date restrictions were applied. Language restrictions were applied to English-language publications only. We additionally screened the reference lists of included articles and relevant reviews and performed forward citation tracking using PubMed.

Search terms combined controlled vocabulary (where available) and free-text keywords across four concept blocks:

1. Depression: “depression”, “depressive symptoms”, “major depressive disorder”, “MDD”, “CES-D”, “BDI”, “PHQ-9”, “MADRS”, “DSRS”, “CDI”, “MINI”
2. Physical activity/exercise: “physical activity”, “exercise”, “training”, “fitness”, “MVPA”, “sedentary”, “inactivity”, “accelerometer”, “walking”, “cycling”
3. Genetics: “genotype”, “polymorphism”, “candidate gene”, “BDNF”, “5-HTTLPR”, “APOE”, “MTHFR”, “SNP”, “polygenic risk score”, “PRS”, “GWAS”
4. Interplay/causal designs: “interaction”, “moderation”, “gene-environment”, “G × E”, “Mendelian randomization”, “instrumental variable”, “two-sample MR”

Reference lists of eligible papers and relevant reviews were also screened to identify additional studies not captured by database searching (backward citation chasing). Where needed, closely related papers from the same cohort were linked and treated as separate studies only if they tested distinct genetic exposures, physical activity operationalizations, or depression outcomes.

### 2.3. Eligibility Criteria

Population. Studies of humans of any age were eligible, including:

- clinical samples with current depression (diagnosis or elevated symptom thresholds),
- community/general population samples assessing depressive symptoms, and
- prospective cohorts assessing incident depression (risk).

Exposure. Eligible studies included at least one physical activity-related exposure, operationalized as:

- structured exercise interventions (any intensity/modality),
- habitual physical activity (self-report or device-based),
- cardiorespiratory fitness (objective tests), and/or
- inactivity or sedentary behavior.

Genetic component. Studies were eligible if they included at least one of:

- candidate–gene variants (single polymorphisms or small panels),
- polygenic risk scores for depression,
- genome-wide genetic susceptibility indices,
- family/twin genetic decomposition relevant to gene–activity correlation or moderation, or
- Mendelian randomization using genetic instruments for activity/inactivity/sedentary behavior and/or depression.

Outcomes. Eligible depression outcomes included:

- clinical depression diagnosis (interview-based or health-record-based),
- incident depression during follow-up,
- depressive symptom severity scales,
- depression treatment response (where exercise was a treatment), and
- depression-related treatment proxies when clearly justified (e.g., antidepressant use), which were interpreted cautiously as indirect outcomes.

Study designs. Randomized controlled trials, non-randomized interventions, cross-sectional and longitudinal observational studies, case-control designs, twin/family designs, and Mendelian randomization studies were eligible.

Exclusion criteria. Studies were excluded if they:

- lacked either a physical activity-related exposure *or* a depression outcome,
- contained genetic information but did not evaluate interplay with physical activity (e.g., genetics-only depression studies),
- examined physical activity and depression without any genetic component,
- were animal/in vitro studies, case reports, editorials, or purely qualitative reports, or
- did not provide sufficient information to extract an effect estimate or direction for a genetics–activity–depression relationship (including interaction, stratified estimates, or causal MR effect).

Only original human studies published as full-text articles in peer-reviewed journals were eligible. Conference abstracts, narrative reviews, editorials, commentaries, protocols, and dissertations were excluded. Studies published in languages other than English were excluded.

### 2.4. Study Selection and Screening

After deduplication, records were screened in two stages: (1) title/abstract screening and (2) full-text assessment. At each stage, two reviewers independently assessed eligibility using predefined criteria. Before formal screening, the reviewers pilot-tested the criteria on a sample of records and refined decision rules to improve consistency. Disagreements were resolved by discussion; when consensus could not be reached, a third reviewer adjudicated. Reasons for exclusion at the full-text stage were recorded and are summarized in the PRISMA flow diagram.

### 2.5. Data Extraction

Data extraction was performed independently by two reviewers using a standardized and pilot-tested extraction form. Discrepancies were resolved by consensus, with arbitration by a third reviewer when required.

A standardized extraction form was used. For each included study, the following were extracted:

- Study characteristics: year, country, design (cross-sectional, cohort, RCT, twin, MR), recruitment frame, sample size, follow-up duration (if applicable).
- Participant characteristics: age range/mean, sex distribution, ancestry/ethnicity restrictions, clinical status at baseline (depressed vs. general population vs. incident-risk sample), and key exclusions.
- Physical activity exposure: measurement method (questionnaire, accelerometer, fitness test), intensity/modality definitions, timing/weekly-pattern definitions (if applicable), and intervention dose/adherence (if applicable).
- Genetic methods: genetic variants (e.g., rs6265/Val66Met; 5-HTTLPR; APOE ε4; MTHFR), genotyping platform (e.g., TaqMan/PCR/array), genetic model (dominant/additive), PRS construction details (if reported), and MR instrument selection thresholds and sensitivity methods (if applicable).
- Depression outcomes: diagnostic system (DSM/ICD), instrument and scoring (e.g., CES-D, BDI, PHQ-9, MADRS), incident-case definitions, and treatment-response definitions (if applicable).
- Analysis and effect sizes: covariates, interaction terms (gene × PA; PRS × PA), stratified estimates, causal MR estimators, and the main quantitative results (OR/HR/RR/β, confidence intervals, *p*-values), emphasizing interaction or moderation evidence where relevant.

When reporting was incomplete or ambiguous, corresponding authors were contacted where feasible; otherwise, the study was retained and the missing item was coded as ‘not reported’. In addition to outcome data, we extracted information on funding sources, declared conflicts of interest, ancestry restrictions, handling of population stratification, multiple-testing correction, and—where relevant—sample overlap between exposure and outcome GWAS in Mendelian-randomization analyses.

### 2.6. Risk of Bias Assessment

Given the heterogeneity of designs, risk of bias was assessed using design-appropriate tools:

- Randomized trials: RoB 2 (randomization process, deviations from intended interventions, missing outcome data, outcome measurement, selective reporting).
- Observational cohort and case-control studies: Newcastle–Ottawa Scale domains (selection, comparability, outcome/exposure ascertainment), supplemented by genetics-specific considerations (population stratification handling, genotyping quality control, multiple-testing control, and plausibility of interaction modeling).
- Cross-sectional studies: appraisal emphasizing sampling/representativeness, measurement validity (especially self-reported activity and symptom scales), and confounding control.
- Twin/family designs: appraisal focused on zygosity determination, within-pair model appropriateness, and interpretation of shared genetic vs. environmental covariance.
- Mendelian randomization studies: ROB-MR/STROBE-MR-aligned domains (instrument strength, sample overlap, horizontal pleiotropy assessment, sensitivity analyses, and directionality checks).

Quality assessments were used to contextualize confidence in findings rather than to exclude studies solely on the basis of score.

### 2.7. Data Synthesis and Presentation

Because included studies differed substantially in populations (clinical vs. community), age (adolescent to older adult), physical activity measurement (self-report vs. accelerometer vs. fitness tests vs. interventions), genetic exposures (single variants, PRS, MR instruments), and depression phenotypes (symptoms, diagnosis, incident cases, treatment response), quantitative meta-analysis was not planned as the primary approach. Instead, findings were synthesized narratively with structured grouping by analytic framework:

1. Candidate–gene interaction studies (e.g., BDNF, 5-HTTLPR, APOE, MTHFR): summarized by whether gene × activity interactions were observed, the context in which they emerged (stress/adversity, sex, symptom dimension, treatment setting), and consistency across studies.
2. Polygenic susceptibility studies: summarized by whether physical activity associations persisted across PRS strata and whether PRS × activity interactions were supported.
3. Lifestyle-context and patterning studies: summarized by activity timing, weekly accumulation patterns, and intensity/modality distinctions, emphasizing analyses that adjusted for total activity volume.
4. Mendelian randomization evidence: summarized by direction of effect (PA → depression; depression → PA), activity measurement (objective vs. self-report), instrument selection, and robustness across sensitivity analyses.

Where multiple reports used the same underlying cohort, results were integrated to avoid double counting while still capturing distinct hypotheses (e.g., timing vs. volume; PRS vs. candidate variants; symptoms vs. diagnosis). The synthesis prioritized (i) consistency across designs, (ii) robustness to confounding control and sensitivity analyses, and (iii) interpretability in the context of gene–environment correlation and bidirectionality.

### 2.8. Registration of Review

The review was registered in the PROSPERO database (identification code: CRD420261398837, Date of creation: 19 May 2026).

## 3. Results

**Figure 1:**
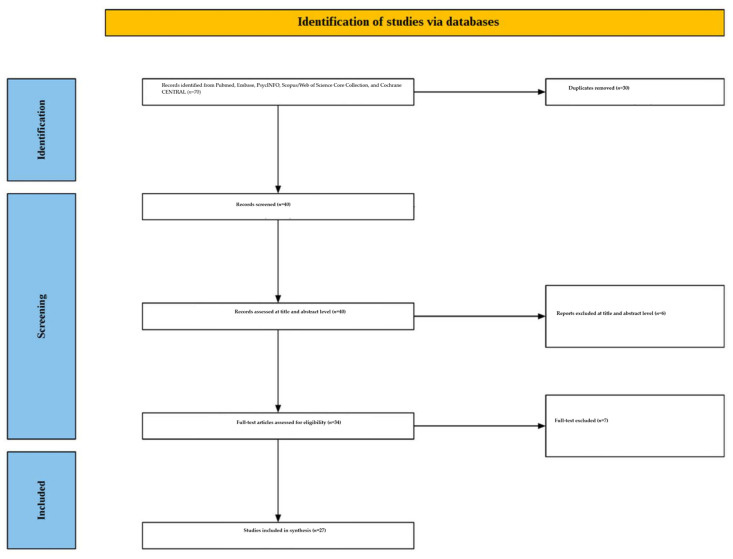
Flow chart depicting the different phases of the systematic review.

Figure 1 provides a summary of the screening process. The initial database search identified 70 studies. After removing duplicates (n = 30), 40 studies were assessed based on their titles and abstracts. Six studies were excluded at this stage. The remaining studies (n = 34) were assessed by full-text review. Seven studies were excluded at this stage. Ultimately, 27 studies were included in the review [17,18,19,20,21,22,23,24,25,26,27,28,29,30,31,32,33,34,35,36,37,38,39,40,41,42,43]. The included studies are presented in Table 1.

### 3.1. Participant Characteristics

Across the 27 studies, participant characteristics were highly heterogeneous, spanning clinical primary-care samples [17], community and registry cohorts [20,21,24,25,27,32,33,35,36,43], student convenience samples [18,26], older-adult intervention cohorts and specialty athletic cohorts [19,22,31], twin and family designs [34,39,40], and very large biobank and GWAS-based datasets used for polygenic and Mendelian randomization analyses [28,37,38,41,42]. Age coverage extended from early adolescence to late adulthood. Adolescent samples included healthy girls aged 10–16 years (n = 82) screened to exclude any current or past Axis I disorder [23], the Dutch population-based TRAILS cohort followed from roughly ages 11 to 16 (genotyped analytic n = 1196) [29], and the Swedish SALVe cohort with repeated waves spanning early adolescence to young adulthood (approximately ages 14, 17, and 20; eligible n = 4712 with wave-specific participation around 890–1337) [30]. Young-adult representation came largely from undergraduate cohorts aged 18–23, both cross-sectional (n = 170) [18] and in a short exercise randomized trial (baseline n = 171; follow-up n = 129) [26]. Midlife was represented by community registries such as the AHAB project (n = 1072; mean age about 44.7 years) [21], while multiple studies focused on older adults: sedentary community-dwelling adults aged 70–89 at risk for mobility disability in LIFE-P [19], cognitively unimpaired adults aged 60–80 in the IPAC exercise trial (n = 99) [22], Taiwanese adults aged 54 and older followed prospectively for six years (eligible n = 639; mean age about 66) [24], and an Austrian cohort of endurance athletes and matched controls aged 60+ (n = 113) [31]. The U.S. Health and Retirement Study diabetes subsample included adults aged 50+ (n = 1051) [32], representing a medically defined subgroup in which functional status, comorbidity, and treatment burden may shape both activity opportunities and depressive symptom expression.

Sex composition varied widely and sometimes reflected selection into the underlying setting. Some population cohorts were near-balanced by sex (e.g., the Spanish PISMA-ep analytic sample of 3123 adults, approximately half women and half men) [20], whereas biobank samples often showed modest female predominance (e.g., Partners Biobank ~57% female) [27]. Several studies examined sex as a central moderator; the LIFE-P genetic subsample included substantially more women than men and sex-stratified patterns were integral to interpretation [19], while other observational analyses also separated results by sex (e.g., PISMA-ep, AHAB, and the HRS diabetes analysis) [20,21,32]. In contrast, certain cohorts were strongly sex-skewed by design or recruitment pool, including the endurance-athlete sample, which was overwhelmingly male (athletes: five women/fifty men; controls: six women/fifty-two men) [31], and the adolescent study restricted to girls only [23]. These distributions matter because sex differences in depression prevalence and physical activity behavior can influence baseline risk, intervention responsiveness, and the detectability of gene-by-activity effects [19,20,21,32].

Clinical status and baseline symptom severity also differed markedly across studies. Only one included sample was explicitly recruited for current depression treatment: Swedish primary-care patients aged 18+ with mild to moderate depression at screening (PHQ-9 ≥ 10) enrolled in a 12-week nonpharmacological treatment trial (physical exercise, internet-based CBT, or treatment as usual), with 547 successfully genotyped participants forming the analytic sample [17]. In contrast, many cohorts were community-based and included participants with a wide range of depressive symptom levels or diagnostic status assessed via interview or records, including DSM-IV MINI diagnoses in a large Spanish epidemiological survey [20] and multiple cohorts using CES-D variants as symptom measures [19,21,24,32]. Several studies—especially student cohorts and older-adult exercise trials—reported generally low baseline symptom levels, highlighting potential floor effects that reduce the room for improvement and can obscure intervention or moderation signals [18,19,22,26]. Other studies sought to reduce reverse causation by excluding baseline depression, for example in large prospective biobank analyses of incident depression defined via health records [27,33,35,36].

Geographic settings were diverse, with data drawn from Sweden [17,30], the United States [19,21,22,32], Spain [20], Taiwan [24,25], Austria [31], the Netherlands [29,39], Denmark [34], Finland [40], and the United Kingdom [28,29,30,31,32,33,34,35,41,42,43]. Recruitment frames ranged from probability-based or population registry approaches (e.g., multistage community sampling in Andalusia, national aging cohorts, and national twin registries) [20,24,32,40] to volunteer registries and health-system or population biobanks (e.g., Partners Biobank and UK Biobank) [27,33,35,36], and to highly selected convenience samples (undergraduate kinesiology students) [18,26] and specialty cohorts (older endurance athletes) [31]. This range implies substantial variation in representativeness and generalizability; for example, student samples are characterized by younger age, higher average physical activity, and lower depression severity [18,26], whereas older-adult cohorts often reflect functional limitations, comorbidity profiles, and in some cases explicit sedentary eligibility criteria [19,22,24,32].

Ancestry and population-structure handling varied and was often most explicit in biobank, polygenic, and MR studies. Several observational genetic analyses restricted samples to reduce confounding by ancestry-related allele frequency differences, including AHAB (non-Hispanic White) [21] and large UK Biobank studies restricted to White British/European or White participants [33,35,36]; the Partners Biobank study similarly analyzed adults of European ancestry [27]. MR studies predominantly relied on European-ancestry GWAS for both exposures and outcomes, drawing heavily on UK Biobank physical activity phenotypes and large psychiatric consortia depression datasets [28,37,38,41,42,43]. Some studies adjusted for genetic principal components in interaction models (e.g., the HRS diabetes analysis) [32]. Conversely, at least one student study explicitly noted that ethnicity was not examined despite known differences in 5-HTTLPR distributions, underscoring uneven attention to population stratification in smaller candidate-gene work [18].

Finally, sample sizes spanned several orders of magnitude, shaping statistical power and inference. Candidate-gene and small intervention studies ranged from n = 82 to about n = 170 [18,22,23,26,31], whereas moderate cohort and epidemiological studies ranged from the mid-hundreds to a few thousand (e.g., Regassa genotyped n = 547 [17]; TRAILS genotyped n = 1196 [29]; PISMA-ep n = 3123 [20]; HRS diabetes n = 1051 [32]; Partners Biobank n = 7968 [27]; SEBAS analytic samples around 600) [24]. Large registry and biobank datasets included tens to hundreds of thousands (e.g., Taiwan Biobank case-control n = 17,934 [25]; UK Biobank prospective analysis n = 339,767 [33]; accelerometer-based timing and weekly pattern analyses n ≈ 76,000–85,000) [35,36]. In MR studies, “participants” consisted of the aggregated samples contributing to GWAS summary statistics—often hundreds of thousands and, for some psychiatric phenotypes, much larger—rather than a single recruited cohort [28,37,38,41,42,43]. Taken together, the reviewed evidence integrates populations differing in age, sex composition, baseline symptom burden, health status, and recruitment representativeness; these differences are central to interpreting heterogeneity in gene–physical activity interplay findings and to judging how well results generalize across life stage and clinical context [17,18,19,20,21,22,23,24,25,26,27,28,29,30,31,32,33,34,35,36,37,38,39,40,41,42,43].

### 3.2. Types of Genetic Tests and Analytic Genetic Frameworks

The included studies used a wide spectrum of genetic approaches, ranging from traditional single-variant “candidate gene” tests to genome-wide polygenic scoring and Mendelian randomization (MR) designs based on large GWAS summary statistics. Broadly, four genetic frameworks were represented: (1) targeted genotyping of specific candidate variants hypothesized to influence neuroplasticity or stress reactivity, (2) polygenic risk scoring to quantify genome-wide susceptibility to depression, (3) cumulative “plasticity” indices combining multiple candidate polymorphisms, and (4) MR using sets of genetic instruments for physical activity, sedentary behavior, and psychiatric outcomes to support causal inference.

Most early studies in the set relied on candidate–gene genotyping, typically assaying one or a small number of functional variants and testing main effects or gene-by-activity interactions. The most frequently studied locus was BDNF Val66Met (rs6265), genotyped from blood or saliva using common laboratory assays (most often TaqMan or related PCR-based methods) and then analyzed under dominant models because Met/Met homozygotes were rare. This approach was used in a depressed primary-care cohort examining response to exercise and internet CBT [17], in large community surveys of depression prevalence and physical activity [20], in a midlife community registry study of depressive symptoms [21], in adolescent cohorts testing gene-by-activity moderation of symptoms [23,30], in older cohorts including an endurance-athlete comparison [31] and a diabetes subsample in the Health and Retirement Study [32], and in intervention trials that included BDNF alongside other candidate markers [19,22]. Several studies also measured BDNF protein (e.g., serum mature BDNF or total serum BDNF), but these biomarker assays were complementary and did not replace the underlying DNA-based genotype testing [17,31]. In practice, candidate–gene work often grouped rs6265 genotypes into Met carriers vs. Val/Val (dominant model) [17,20,21,23,31,32], though some adolescent analyses retained three genotypes (GG/GA/AA) when sample size permitted [30].

A second commonly used candidate polymorphism was the serotonin transporter promoter length variant 5-HTTLPR, genotyped using PCR amplification and fragment-length discrimination (classifying short vs. long alleles). This variant was examined in a cross-sectional moderation study in undergraduates [18], in an exercise intervention trial evaluating genotype moderation of symptom change [26], and as part of the LIFE-P older-adult trial alongside BDNF and APOE [19]. Because triallelic functional classification (e.g., LA/LG subdivision) was not consistently implemented, several studies relied on the simpler biallelic s/L model and, when needed for power, collapsed categories into l-carriers vs. ss [18,26]. Candidate testing also extended beyond BDNF and 5-HTTLPR to variants indexing broader neurobiological risk. APOE ε4 status was used as a moderator in older-adult intervention and cohort contexts [19,22,24], and one large biobank-linked case-control study examined an MTHFR variant (rs17367504) with exercise effect modification [9]. These candidate–gene studies typically employed standard quality-control checks (e.g., call rates, Hardy–Weinberg equilibrium, blind genotyping procedures) and analyzed genotype effects as main effects and interactions with physical activity exposure, intervention assignment, or contextual stressors [17,18,20,23,24,25,26,30,31,32].

Beyond single variants, one longitudinal adolescent study operationalized candidate variation within a “genetic plasticity” framework, constructing a cumulative index across multiple polymorphisms often discussed in differential susceptibility (including serotonergic, dopaminergic, and other loci, with BDNF included) and then testing whether the physical activity–depression reciprocity differed across low/intermediate/high plasticity groups [29]. This approach differs from polygenic risk scoring in that it combines a small, theory-chosen set of variants rather than genome-wide discovery-based weights, and it is typically interpreted as indexing sensitivity to environments rather than additive disease risk.

In contrast to these targeted strategies, several more recent studies used genome-wide information to quantify vulnerability using polygenic risk scores (PRS) for depression. Here, individual-level genotype data were used to compute a weighted sum of many SNPs across the genome, typically based on large external GWAS summary statistics and then standardized within the analytic cohort. PRS-based designs were used to test whether physical activity protects against incident depression even in those at high genetic risk, and whether associations vary across PRS strata [27,33,35,36]. Compared with candidate-gene work, PRS approaches aim to better reflect the highly polygenic architecture of depression and typically have greater predictive validity for risk gradients, although they remain ancestry-sensitive and depend on the discovery GWAS used to derive weights.

Finally, multiple studies leveraged genetic data not as a vulnerability marker but as a quasi-experimental tool for causal inference using Mendelian randomization. In these analyses, sets of SNPs associated with exposures such as accelerometer-measured activity, self-reported activity, specific activity types, sedentary behavior, or sleep were selected as instruments from large GWAS, LD-clumped, and then related to depression or psychiatric outcomes in independent GWAS datasets [28,37,38,41,42,43]. Several MR studies were explicitly bidirectional, testing both PA → depression and depression liability → PA (and related behaviors), and some compared instrument performance across objective versus self-report activity phenotypes, often finding stronger or more consistent evidence when activity was measured objectively [28,37,38]. MR studies typically supplemented primary inverse-variance weighted estimates with sensitivity estimators (e.g., weighted median, MR-Egger) and pleiotropy/outlier diagnostics (e.g., MR-PRESSO), reflecting a focus on instrument validity and robustness [28,37,38,41,42,43]. Collectively, these MR designs shift the genetic question from “who is most susceptible?” to “is the activity–depression relationship plausibly causal, and in which direction?”, complementing observational gene-by-activity interaction findings.

### 3.3. A Convergent Finding: Physical Activity Shows Protective Associations Across Genetic Risk Frameworks

A consistent result across several methodological traditions in this review is that higher physical activity (or lower inactivity) is associated with lower depression risk or symptom burden, and this pattern often persists even when genetic liability is explicitly modeled. This convergence is most clearly illustrated in studies using genome-wide approaches—polygenic risk stratification and Mendelian randomization—because these designs reduce reliance on single-variant effects and, in the case of MR, are specifically aimed at strengthening causal interpretation.

In the polygenic framework, evidence from large cohorts indicates that physical activity is protective across the genetic-risk spectrum rather than only in genetically “vulnerable” subgroups. In the Partners Biobank analysis, depression polygenic risk score (PRS) predicted higher odds of incident depression over two years, while baseline physical activity predicted lower odds, and the two were essentially uncorrelated—supporting the interpretation that activity is not merely a proxy for lower genetic liability [27]. When participants were stratified into low, intermediate, and high PRS categories, higher physical activity remained associated with reduced incident depression in every stratum, and the high-PRS participants who were more active had an incidence similar to low-PRS participants who were less active, illustrating that behavioral exposure can meaningfully offset inherited risk at the population level [27]. Similar “independent contributions” were observed in a larger UK Biobank prospective analysis using a composite healthy-lifestyle score that included meeting physical activity guidelines: healthier lifestyle profiles were associated with lower incident depression across PRS groups, and formal interaction testing did not support the idea that the lifestyle–depression association differed substantially by genetic risk [33]. Together, these PRS-based results support an additive rather than strongly multiplicative interpretation: genetic susceptibility elevates baseline risk, but activity-related behaviors remain relevant across strata.

Genetic causal-inference studies point in the same direction, particularly when physical activity is measured objectively. In bidirectional two-sample MR, accelerometer-based physical activity instruments provided evidence consistent with a causal protective effect of activity on major depressive disorder, whereas self-reported activity instruments were less consistent—suggesting that measurement error in self-report may weaken instruments and obscure effects [28]. Related MR work using accelerometer phenotypes also supported a protective causal role of physical activity for depression and depression severity, alongside evidence that depression liability can causally reduce activity (a plausible feedback loop that complicates observational interpretation) [37]. Importantly, the MR pattern does not imply that all activity phenotypes behave identically: across studies, protective effects were more reproducible for global or objectively measured activity traits than for some self-report domains, underscoring that “physical activity” is not a single exposure but a family of behaviors with different measurement properties and genetic architectures [28,37,38].

Prospective accelerometer-based cohort analyses further extend this result by showing that not only total volume but how activity is accumulated can relate to subsequent depression risk, again without strong evidence that effects are confined to specific genetic-risk subgroups. For example, UK Biobank analyses found that meeting guideline-level MVPA was associated with lower incident depression risk whether accumulated as “weekend warrior” bouts or more regularly across the week [36]. Another accelerometer study reported that earlier timing of total daily activity (e.g., early-morning peaks) and morning or midday–afternoon MVPA were associated with lower incident depression risk after accounting for total activity volume; while results varied somewhat across PRS strata in stratified models, the overall interaction evidence was limited, aligning with the broader theme that activity patterns matter but do not appear to confer benefit only among those with particular genetic profiles [35].

Taken together, these findings support a central inference for the gene–physical activity interplay literature: genetic risk for depression is not destiny in the face of modifiable behavior, and physical activity shows protective associations that are visible in multiple analytic frameworks, including those explicitly designed to account for genetic confounding or reverse causation [27,28,33,35,36,37]. At the same time, the convergence is not perfect. Candidate-gene moderation results were often context-dependent—emerging in specific sex strata, stress environments, or symptom dimensions—and smaller studies frequently had low baseline symptoms that may have limited power to detect interactions or change [18,19,22,23,26,30]. Thus, the strongest “one-result” takeaway from the current evidence base is not that a single genotype reliably determines who benefits most, but that physical activity generally tracks with lower depression risk even when genome-wide genetic susceptibility is considered, and that objective measurement and prospective or MR designs tend to provide the clearest support for this protective relationship [27,28,33,35,36,37].

### 3.4. Context-Dependent Moderation in Candidate-Gene Studies: Effects Cluster in Specific Subgroups and Exposures

In contrast to the broadly consistent “activity is protective” pattern observed in polygenic and MR analyses, the candidate–gene literature in this review most often yielded context-dependent results, where genetic moderation emerged only under particular conditions—such as specific developmental windows, stress exposures, symptom dimensions, or sex strata—rather than as stable main effects. A clear example is the Swedish SALVe cohort analysis of BDNF rs6265 (Val66Met), which reported no overall genotype differences in depressive symptoms across waves but detected moderation effects that were wave-specific and environment-contingent [30]. In early adolescence (wave 1), physical activity interacted with genotype in predicting depressive symptoms: activity moderated the association between rs6265 and both continuous symptom counts and the binary depressive-symptom classification, with genotype differences most evident at very low levels of activity [30]. By mid-adolescence (wave 2), childhood stress—measured as a parent-rated overall stress indicator—became the more salient moderator, with evidence that AA carriers showed a “for-better-and-for-worse” pattern: lower symptoms at very low stress and higher symptoms at elevated stress [30]. By young adulthood (wave 3), these interaction signals were not detected, which the authors interpreted in light of reduced sample size and potentially changing developmental dynamics [30]. This pattern underscores a central theme in gene–environment interplay: genotype effects may not generalize across age or context, and the same polymorphism can appear irrelevant in main-effect tests yet show meaningful moderation in narrowly defined conditions.

A similarly contingent moderation signal appears in the Swedish primary-care trial cohort nested within the Regassa study, where BDNF Val66Met interacted with childhood adversity (CA) to predict treatment response, with the interaction most pronounced in the physical exercise arm [17]. Here, genotype alone did not predict response, and CA alone did not predict response, but their interaction did: among participants reporting no CA, Met carriers had higher odds of responding (≥50% reduction in clinician-rated MADRS), whereas among Met carriers exposed to mild–moderate or high CA, response likelihood was substantially reduced [17]. When stratified by treatment type, the pattern was strongest for physical exercise, where the apparent Met-carrier advantage was largely confined to those without CA; the same interaction pattern was weaker and non-significant in the internet CBT arm and not evident in treatment as usual [17]. Importantly, parallel analyses of circulating BDNF (mature BDNF and proBDNF) did not explain the clinical pattern, suggesting that the moderating effect—if real—was not mediated by the measured serum markers in this dataset [17]. Together, SALVe and Regassa illustrate that candidate-gene moderation often appears only when the behavioral exposure is embedded in a broader “lifestyle context” (e.g., activity plus stress history, or activity as a treatment in clinical depression), rather than as a direct genetic determinant of depression outcomes.

These context-bound findings help explain why the candidate–gene literature is mixed across studies. Samples differed sharply in baseline symptom severity (often low in nonclinical cohorts), exposure measurement (self-reported activity frequency vs. structured exercise interventions), and subgroup composition (sex balance, age range, adversity prevalence), all of which can alter power and the plausibility of detecting interaction effects [17,18,22,23,26,30]. Notably, the developmental specificity observed in SALVe—activity moderation early, stress moderation later—also aligns with the broader point that adolescence is not a single uniform exposure window, and that the salience of physical activity and psychosocial stress may shift across puberty, school transitions, and emerging adult roles [30]. Overall, the candidate–gene results in this review are best summarized not as evidence for a single “activity-response genotype,” but as evidence that genetic moderation—when observed—tends to be conditional on developmental timing, stress/adversity context, and symptom domain, and therefore may be difficult to replicate unless those contextual features are closely matched across studies [17,30].

### 3.5. Genetic Moderation Is Often Absent: Null Interaction Findings in Larger or More Stringent Designs

A noteworthy result across the reviewed literature is that many studies explicitly testing gene × physical activity (or exercise) interactions reported null or weak moderation, even when main effects of activity or genotype were present. These null findings are informative because they suggest that, for several commonly studied candidate variants, any differential “susceptibility” to the mental-health effects of activity is either small, highly context-specific, or difficult to detect with typical measurement and sample constraints.

Several studies focusing on BDNF Val66Met (rs6265) found no evidence that genotype reliably changed the association between physical activity and depressive symptoms. In the midlife AHAB cohort (n = 1072), physical activity and depressive symptoms showed sex-specific associations (clearer inverse associations in women), and Met-carrier status related to higher depressive symptoms in men, yet the critical BDNF × activity interaction term was not significant in either linear models of CES-D scores or logistic models predicting clinically significant symptoms (CES-D ≥ 16) [21]. Similarly, in the Health and Retirement Study diabetes subsample (n = 1051), physical activity was inversely associated with depressive symptoms in both genotype strata, but formal tests indicated no significant moderation of the activity–depression association by BDNF status; subgroup contrasts by sex suggested differences in magnitude, yet genotype-by-activity interactions remained non-significant [32]. In the Spanish PISMA-ep epidemiological study (n = 3123), physical activity indicators were associated with lower odds of DSM-IV major depression, and an interaction emerged specifically for hours of activity, but there was no significant interaction for simpler activity measures (active vs. inactive) or for intensity categories, underscoring that moderation signals may be sensitive to how “dose” is operationalized and may not generalize across exposure definitions [20]. Taken together, these results suggest that even for a biologically plausible locus like BDNF, interaction evidence is inconsistent: some designs detect moderation only under particular operationalizations or contexts, whereas others find none [20,21,32].

Null moderation effects were also common in exercise-intervention contexts where randomization strengthens causal inference about exercise assignment. In the IPAC randomized trial of older adults (n = 99), neither BDNF Val66Met nor APOE ε4 moderated exercise effects on depressive symptoms, and the overall randomized comparisons did not show robust changes in depression, anxiety, or stress, likely reflecting low baseline symptom levels and limited power for genetic subgrouping [22]. In the older-adult LIFE-P trial, average depressive symptom reduction did not differ significantly between the physical activity intervention and health-education control when moderation was ignored, and the strongest signals appeared only for specific symptom dimensions (notably somatic symptoms) and sex-by-genotype patterns; APOE ε4 showed no meaningful association with symptom change in these analyses [19]. These intervention findings highlight an important distinction: even when structured exercise is delivered, genetic moderation may not manifest as a broad change in overall depression scores, and where signals appear they may be dimension-specific or sex-specific rather than reflecting a general “exercise responder genotype” [19,22].

Longitudinal and genetically informative observational designs further reinforce the frequency of null moderation. In the TRAILS adolescent cohort, cross-lagged panel modeling supported small reciprocal prospective links between physical activity and depressive symptoms across adolescence, yet neither a cumulative “genetic plasticity” index nor any individual candidate polymorphism (including BDNF and 5-HTTLPR) significantly moderated these reciprocal effects under the authors’ stricter multiple-testing threshold [29]. This suggests that, at least in that population-based adolescent sample and modeling framework, genetic moderation of the PA–depression linkage—if present—is unlikely to be large or consistent across time. Similarly, twin and family designs emphasized that the exercise–distress association can be largely explained by shared underlying factors. In the Netherlands Twin Register analysis, cross-sectional and longitudinal associations between leisure-time exercise and anxious/depressive outcomes were small and appeared to reflect genetic covariance rather than environmental coupling; within monozygotic twin pairs, differences in exercise did not reliably predict differences in symptoms, and within-person changes in exercise did not track symptom changes over years, arguing against a strong direct causal effect of exercise on distress at the population level as measured in that dataset [39]. While this does not directly test a specific SNP-by-activity interaction, it is highly relevant to “genetic moderation” claims because it implies that genetic factors jointly influencing exercise behavior and emotional distress can generate associations that resemble protective effects without requiring strong environmentally driven (behaviorally modifiable) covariance [39].

Overall, these null or weak moderation findings serve as a counterweight to more striking subgroup interactions observed in selected contexts. They suggest that for widely studied candidate variants, robust moderation is not the norm; rather, detectable effects tend to depend on particular symptom domains, measurement choices (e.g., activity “dose” vs. participation), baseline symptom distributions, and sample size for genetic subgrouping [19,20,21,22,29,32,39]. In synthesis, the evidence supports caution in interpreting isolated candidate-gene interaction findings as generalizable, and it reinforces the value of large-scale polygenic and MR frameworks for understanding how genetic liability and physical activity relate to depression at the population level [27,28,33,37,38].

### 3.6. A Distinct Interaction Pattern: Activity Timing and Concentration Relate to Incident Depression Beyond Total Volume

Beyond “how much” activity people do, one of the more novel results in this literature is that when activity is accumulated—across the day or across the week—can be associated with later depression risk, even after accounting for total activity volume. Two large accelerometer-based UK Biobank studies highlight this pattern and suggest that behavioral “activity architecture” may matter for depression prevention, with only limited evidence that these timing effects are strongly contingent on genetic risk.

First, in the accelerometer timing study, participants were clustered by the daily timing of total physical activity (TPA) and categorized by the timing of MVPA (among those meeting activity thresholds). After excluding prevalent depression and adjusting for overall activity volume and other covariates, an early-morning peak in total daily activity was associated with a lower hazard of incident depression compared with a midday–afternoon reference pattern, and MVPA concentrated in the morning (and in some analyses midday–afternoon) was also associated with reduced risk versus inactivity [35]. Although the authors explored stratification by depression polygenic risk score (PRS), results did not indicate a strong multiplicative timing × PRS interaction; instead, the main impression was that earlier activity timing can be beneficial across the cohort, with some variation in which timing categories reached statistical significance within PRS strata [35]. This suggests that timing may operate as an additional behavioral dimension of relevance to depression risk rather than merely a proxy for “more active” individuals.

Second, a separate UK Biobank accelerometer analysis addressed whether the weekly pattern of MVPA accumulation matters, comparing “weekend warriors” (≥150 min/week MVPA, ≥50% in 1–2 days) with “active regular” participants (≥150 min/week spread more evenly). Over long-term follow-up, both active patterns showed similarly lower risks of incident depression compared with inactive participants, and the weekend-warrior pattern was not meaningfully worse than regular accumulation when total MVPA met guideline levels [36]. This result is important because it implies that, for depression risk, meeting the overall MVPA target may be more consequential than whether activity is evenly distributed, potentially lowering a practical barrier to behavior change for people who can only be active on limited days.

Together, these findings extend the gene–physical activity interplay discussion in two ways. First, they shift focus from genotype-specific responsiveness to behavioral patterning that may influence mood-relevant physiology (e.g., circadian alignment, sleep–wake regularity, or daytime light exposure) without requiring differential genetic susceptibility to be clinically meaningful [35,36]. Second, because these studies used objective accelerometer measures, they reduce concerns about self-report bias and provide higher-resolution exposure characterization than most candidate-gene interaction work [35,36]. In synthesis, the accelerometer evidence suggests that depression risk may be sensitive not only to total activity volume but also to temporal distribution, with earlier daily activity and flexible weekly accumulation (including weekend-warrior patterns) both compatible with lower incident depression risk, and with limited indication that these benefits are restricted to particular genetic-risk groups [35,36].

### 3.7. Exercise Intensity and Modality: Limited Evidence That “Harder Is Better,” with Occasional Genotype-Specific Signals

Another recurring result in this review is that exercise intensity and modality do not show a uniformly graded relationship with depression outcomes, and when intensity-related effects appear, they are sometimes small, subgroup-specific, or inconsistent across measurement approaches. This nuance matters because “physical activity” is operationalized very differently across studies—ranging from light-intensity lifestyle movement to structured high-intensity training—and these differences shape both average effects and the plausibility of detecting gene-by-activity interplay.

In large community data, activity intensity was not consistently associated with lower depression risk in a simple dose–response manner. In the Spanish PISMA-ep epidemiological sample, reporting any physical activity was associated with lower odds of DSM-IV major depression, but the most consistent association was observed for light-intensity activity, whereas moderate intensity was borderline and vigorous activity was not clearly protective in that cohort [20]. This pattern cautions against assuming that higher intensity necessarily yields stronger mental-health benefits in population settings, particularly when intensity is self-reported and may correlate with factors such as health status, occupational strain, or training behaviors that are unevenly distributed across the population [20].

Intervention evidence similarly challenges a straightforward “more intense is better” conclusion. In the IPAC randomized trial comparing high-intensity interval cycling, moderate-intensity continuous cycling, and a no-contact control in older adults, there were no overall between-group differences in changes in depression, anxiety, or stress over six months, and BDNF Val66Met did not moderate mental-health response; the authors emphasized low baseline symptom levels as a likely constraint on detecting change [22]. The main genotype-related signal in that study emerged not for depression but for perceived stress, where APOE ε4 carriers assigned to high-intensity training showed reductions in stress, suggesting that intensity effects—when detectable—may be outcome-specific rather than broadly antidepressant [22]. In LIFE-P, a year-long multi-component physical activity program did not reduce total depressive symptoms on average compared with health education, but it did show more favorable change for somatic symptom dimensions in men, with a marginal BDNF-by-sex-by-intervention pattern—again pointing to domain- and subgroup-specific benefits rather than a global intensity-driven effect [19].

Observational designs also highlight that “high activity” can reflect qualitatively different phenomena depending on the population. In a student moderation study of 5-HTTLPR, the sample was unusually active, and the authors interpreted a counterintuitive pattern at very high activity levels as possibly reflecting overtraining-related symptoms rather than a protective effect of high volume [18]. While this interpretation is context-dependent and not definitive, it illustrates an important point for synthesis: in certain groups, extremely high activity may be correlated with stress, injury, sleep disruption, or performance pressures that could attenuate or reverse expected mood benefits [18].

Taken together, these results suggest that the relationship between intensity/modality and depression is not reliably monotonic, and genetic moderation—where observed—tends to be outcome- or subgroup-specific rather than a consistent “high-intensity responder” profile. Across studies, light-to-moderate activity often appears at least as relevant as vigorous activity for depression-related outcomes in real-world cohorts, whereas trials contrasting intensity levels sometimes fail to show clear differential mental-health benefits, especially when baseline symptom levels are low [19,20,22]. This pattern supports a pragmatic inference for the broader review: genetic susceptibility may shape some individual differences in response, but the available evidence does not strongly justify prioritizing intensity escalation as a universally superior strategy for depression outcomes compared with increasing achievable activity volume and regularity.

### 3.8. Familial Confounding and Gene–Environment Correlation: Evidence from Twin Designs and Within-Family Comparisons

A further result that remains important for interpreting gene–physical activity interplay is that part of the observed activity–depression association can reflect shared familial factors (including genetics) rather than a direct environmental effect of activity itself, yet some within-family analyses still suggest a protective signal that is not fully explained by shared background. This is most clearly demonstrated by twin and family designs, which are specifically structured to separate causal interpretations from genetic or family-environment confounding.

In the Netherlands Twin Register family study, leisure-time exercise showed small negative associations with depressive and anxiety-related traits at the population level, but the pattern of covariance was dominated by genetic overlap rather than environmental coupling: bivariate genetic models indicated significant genetic correlations between exercise and distress-related outcomes, while environmental correlations were not clearly different from zero [39]. Crucially, more direct quasi-causal tests did not support a strong causal interpretation in that dataset: within monozygotic twin pairs, the twin who exercised more did not reliably report fewer symptoms than their genetically identical co-twin, and within-person increases in exercise over time did not consistently track decreases in symptoms [39]. This constellation of results implies that, at least in that sample and exposure definition (leisure-time exercise), the association may be partly generated by gene–environment correlation—genetic propensities that jointly influence both likelihood of exercising and vulnerability to distress—rather than exercise causally reducing symptoms in a way detectable through within-family contrasts [39]. For the broader review, this is a key caution: candidate–gene or observational moderation findings can be difficult to interpret if the exposure (physical activity) is itself genetically influenced and correlated with mood-related predispositions.

However, the Finnish Twin Cohort co-twin control study provides a complementary result suggesting that persistent activity may still have protective relevance even after controlling for shared genes and upbringing, at least for certain depression-related endpoints. By comparing twins strongly discordant for long-term leisure-time physical activity across adulthood and then examining later antidepressant purchases from national prescription registers, the study found that the more active twin had lower odds of any antidepressant use during follow-up in within-pair analyses [40]. The association was evident across discordant pairs overall and remained directionally similar in monozygotic pairs, strengthening in fully adjusted monozygotic models despite limited precision due to smaller numbers [40]. Notably, the protective association was clearest for “any use” and for more occasional use patterns, whereas evidence was weaker for sustained long-term antidepressant purchasing, suggesting that activity may be more strongly related to milder or intermittent treatment trajectories than to chronic or recurrent courses captured by repeated purchases [40]. Although antidepressant use is an imperfect proxy for depression incidence or severity, the use of objective register data and within-pair contrasts provides meaningful leverage against confounding explanations and indicates that at least part of the activity–depression relationship may not be fully reducible to shared familial factors [40].

Together, these two results sharpen the interpretation of gene–physical activity interplay. First, they support the view that physical activity is genetically influenced and that genetic factors can confound observational associations with depression outcomes, which helps explain why simple gene × activity findings can be inconsistent or difficult to replicate across cohorts [39]. Second, they also suggest that when activity is measured as a stable, long-term behavioral pattern and outcomes are captured objectively over time, a within-family protective signal can still emerge, implying that causality is plausible in at least some contexts even under stringent control for familial background [40]. In synthesis, twin and within-family evidence in this review argues for both caution and nuance: some activity–depression associations may reflect shared genetic liability, but persistent activity may still confer measurable protection against downstream depression-related treatment markers in ways not fully explained by shared genes or upbringing [39,40].

### 3.9. Depression Liability Can Reduce Physical Activity: Bidirectional Evidence for a Feedback Loop

A remaining result worth highlighting is that several genetics-based causal analyses support bidirectionality, particularly the idea that depression risk is not only influenced by activity, but can also causally suppress physical activity, creating a feedback loop that complicates interpretation of observational associations. This matters for the gene–physical activity interplay question because gene-by-activity findings can be distorted if baseline or emerging depressive symptoms reduce activity levels (reverse causation), and because genetically informed evidence can help separate “activity protects” from “depression reduces activity” pathways.

In bidirectional Mendelian randomization work using large European-ancestry datasets and objective accelerometer phenotypes, there was evidence that higher physical activity causally lowers depression risk and severity, but also that greater genetic liability to depression causally predicts lower physical activity [37]. This reverse-direction effect was interpreted as consistent with clinically plausible mechanisms—anhedonia, fatigue, psychomotor slowing, sleep disruption, and reduced motivation—that can reduce engagement in physical activity. Importantly, the same analysis did not provide parallel evidence that depression liability increases sedentary time to the same extent, suggesting that depression-related behavioral change may manifest more strongly as reduced active behavior than as uniformly increased sitting across all contexts [37]. Complementing this, another bidirectional MR study examining inactivity constructs based on UK Biobank questionnaire items reported that genetic liability to major depressive disorder predicted slightly lower participation in several activities and slightly higher odds of inactivity, consistent with depression risk nudging individuals away from activity engagement [41]. Although these reverse-direction effects were often smaller in magnitude than the forward activity → depression estimates, their consistency across multiple activity/inactivity definitions supports a “vicious cycle” framing in which depression predisposition and low activity can reinforce each other [37,41].

This bidirectional result helps contextualize why self-report activity measures sometimes behave inconsistently across causal analyses. In two-sample MR, evidence for a protective causal effect of physical activity was clearest when activity was measured objectively (accelerometer-based), whereas self-reported activity instruments often produced null or unstable estimates [28]. One interpretation is that self-report activity is more susceptible to mood-related reporting bias and to behavioral suppression driven by subclinical depressive symptoms, both of which can weaken genetic instruments and blur directionality. If depression liability reduces activity, then cross-sectional associations between low activity and higher symptoms can reflect both causal directions simultaneously, making it difficult for simple observational gene-by-activity interaction models to cleanly isolate moderation effects.

Overall, the evidence summarized here implies that gene–physical activity interplay should be interpreted in a dynamic rather than one-way framework. Physical activity may lower depression risk, but depression liability can also reduce activity, meaning that interventions may need to address both sides of the loop—supporting activity initiation/maintenance while also recognizing that emerging depressive symptoms can be a barrier to being active [28,37,41].

### 3.10. “Dose” and Measurement Matter: Interaction Signals Often Depend on How Physical Activity Is Operationalized

A final result that remains important to synthesize is that gene–physical activity interplay findings are frequently sensitive to the specific physical activity metric used—hours versus frequency, participation versus intensity, self-report versus accelerometer, and composite “total activity” versus specific activity domains. Across studies, moderation signals were more likely to appear for some operationalizations than others, and null findings in one metric often coexisted with effects in another, underscoring that measurement choices can determine whether gene-by-activity effects are detectable.

This dependence on operationalization is well illustrated in the large Spanish PISMA-ep study of BDNF Val66Met. Physical activity assessed as “any activity” (yes/no) and activity intensity categories showed clear main associations with lower depression prevalence (particularly for light intensity), yet the BDNF interaction signal emerged specifically for hours per week of activity: as hours increased, Met carriers showed lower odds of depression relative to Val/Val individuals, while interactions with the simpler activity measures were not significant [20]. In other words, the same dataset contained (i) robust activity–depression associations, (ii) null main genetic effects, and (iii) an interaction detectable only when “dose” was modeled in a more continuous way [20]. This pattern suggests that binary participation indicators may be too coarse to capture differential benefit by genotype, whereas “dose” measures can sometimes reveal gradients—though at the cost of greater susceptibility to reporting error.

A related measurement lesson emerges when comparing self-report and objective activity in causal genetic designs. In two-sample Mendelian randomization, protective effects of activity on depression were supported when activity was indexed by accelerometer-derived phenotypes, whereas self-reported activity instruments showed weaker or null evidence, despite much larger sample sizes for the self-report GWAS [28]. This implies that precision and validity of the exposure measure can matter more than sheer N: measurement noise in self-report can weaken genetic instruments and obscure true effects. Parallel conclusions appear in broader MR work that similarly relied on accelerometer phenotypes for stronger inference about activity and depression outcomes [37,38]. Cohort studies using accelerometry extended this point by demonstrating that more granular exposure constructs—such as timing of activity across the day or concentration across the week—predict incident depression risk even after adjusting for total volume, a nuance that would be difficult to detect with standard questionnaire measures [35,36].

Candidate–gene studies in small samples also demonstrate how different activity metrics can yield different interaction patterns. In adolescents, activity measured as a general self-report activity level (PAQ-CA) was sufficient to detect a BDNF-by-activity interaction in girls, but the authors also noted that alternative derived subcomponents (e.g., activity frequency indices) produced similar conclusions, suggesting that even within self-report, certain activity representations may better capture the behavior relevant to mood than others [23]. In student samples, very high estimated energy expenditure created interpretive ambiguity (e.g., possible overtraining), illustrating that “high activity” can mean different things depending on population context and the scale used [18]. Even in intervention studies with controlled exercise prescription, mental-health effects could differ by the outcome scale and symptom dimension assessed (e.g., somatic symptoms versus total depressive symptoms), reinforcing that “what changes” can depend on measurement at the outcome level as well [19,22].

Overall, these results imply that inconsistency across gene–physical activity studies is not only about genetic model choice or sample size; it is also a function of exposure measurement. Coarse self-report metrics (active vs. inactive) may detect broad main effects but miss moderation; continuous or domain-specific measures may reveal interaction gradients but increase susceptibility to bias; and objective accelerometer measures tend to provide clearer, more reproducible signals for both causal inference and refined behavioral patterns [20,23,28,35,36,37,38]. For the broader review, this supports treating “physical activity” as a family of related but non-equivalent exposures and interpreting gene-by-activity findings in light of the specific operationalization used.

### 3.11. Risk of Bias

The risk of bias assessment is presented in Table 2 (RoB-2) and in Table 3 (ROBINS-I).

## 4. Discussion

Across the included studies, the most consistent message is not that any single “depression gene” determines whether physical activity helps, but that depression–activity links are (i) modest on average, (ii) highly context dependent, and (iii) more reliably detected when activity is measured well (e.g., accelerometry) and genetic liability is modeled polygenically rather than as single candidate variants. This helps reconcile why some candidate–gene studies report striking subgroup effects while others find null moderation: exercise can be antidepressant overall, yet the size and detectability of benefit varies with baseline symptom severity, stress history, sex, medication status, activity dose/intensity, and measurement error.

### 4.1. Why the Findings Range from “Clear Moderation” to “No Moderation”

A wide spread of results is exactly what you would expect when (i) the average antidepressant signal of physical activity is real but modest, (ii) “depression” is biologically and clinically heterogeneous, and (iii) gene–environment (G × E) moderation effects are usually small, highly context-dependent, and statistically fragile unless samples are very large and measurement is strong. Contemporary randomized-trial evidence indicates that exercise can reduce depressive symptoms meaningfully on average, but with substantial between-study and between-person heterogeneity (i.e., some people improve a lot, some a little, some not at all). A large network meta-analysis of randomized controlled trials in BMJ concluded that exercise is effective for depression, but also emphasized variation by exercise modality, intensity, and study features—exactly the kind of heterogeneity that can make subgroup/moderation signals appear in some designs and vanish in others [4].

#### 4.1.1. Moderation Is Easier to “See” When the Environment/Treatment Actually Moves the Biology You Are Testing—And When That Biology Is on the Causal Path

Candidate variants such as BDNF Val66Met are often chosen because the underlying protein is mechanistically plausible: BDNF supports synaptic plasticity and hippocampal function, and the Met allele alters activity-dependent trafficking/secretion in ways that can influence neural circuit responsivity to experience. Classic human neuroimaging and molecular work shows that Val66Met relates to hippocampal processing and that the Met allele can change neural responses in memory-related circuits—evidence that the variant can matter, but not necessarily in a simple “main effect on depression” way [44,45]. However, whether exercise engages that same pathway depends on training dose, adherence, and timing relative to stress exposure. Peripheral BDNF changes after exercise are often transient and sensitive to assay choices and platelet release (serum vs. plasma), which makes “BDNF as the mediator” difficult to verify using resting blood draws. Reviews and meta-analytic work show that acute exercise can increase peripheral BDNF, while chronic training effects on resting BDNF are inconsistent and may be small or absent in some clinical contexts—so it is unsurprising that several included studies found genotype patterns in symptoms without parallel explanatory shifts in serum BDNF [46,47].

#### 4.1.2. “Depression” Varies Across Studies in Ways That Change What Counts as a Response—And Which Mechanisms Are Likely to Matter

Across the included literature, outcomes range from incident clinical depression in health-record cohorts to symptom scales in community samples to subdimensions (somatic vs. affective) in older adults. Those are not interchangeable phenotypes. Somatic symptoms (sleep, fatigue, appetite, psychomotor changes) can be more tightly linked to inflammation, metabolic disease, and physical deconditioning—domains where exercise has direct physiological leverage—whereas cognitive–affective symptoms may be more sensitive to psychosocial context or psychotherapy content. Mechanistically, exercise influences multiple systems plausibly relevant to different depressive “subtypes”: anti-inflammatory signaling (including exercise-induced myokines and downstream cytokine modulation), neurotrophin and synaptic-plasticity pathways, HPA-axis regulation, and peripheral-to-central metabolic signaling [48,49].

A concrete example of pathway specificity is the kynurenine pathway: exercise-induced skeletal-muscle adaptations (via PGC-1α1) can shift kynurenine metabolism in ways that promote resilience to stress-induced depressive-like behavior in experimental models. This supports a biologically credible route by which “the same exercise dose” might produce different mood effects depending on baseline metabolic/inflammatory state—helping explain why moderation signals sometimes appear in subgroups with particular vulnerabilities (e.g., chronic stress, metabolic illness) but not in healthier, low-symptom samples [9].

#### 4.1.3. Measurement Differences (Especially Self-Report vs. Objective Activity) Can Flip Results from “Signal” to “Null”

A recurring pattern in the broader literature is that objective accelerometer activity often yields clearer causal/longitudinal inference than self-report. Self-reported physical activity contains substantial error (recall bias, social desirability, “rounding,” and misclassification of intensity), which attenuates associations and makes interaction terms even harder to detect because interactions are effectively “second-order” effects. When exposure measurement is noisy, moderation estimates are biased toward zero, and power collapses. This is one reason Mendelian randomization studies and large cohorts often show stronger evidence when activity is accelerometer-derived than when it is questionnaire-derived [9,47].

The implication for the included candidate–gene moderation studies is straightforward: a true genotype-by-activity interaction could exist biologically, yet look null when activity is measured with broad self-report instruments (or when only “hours/week” are captured without intensity, patterning, or adherence).

#### 4.1.4. Many “Strong Moderation” Findings Arise in Precisely the Conditions That Inflate Apparent Effect Sizes: Small Samples, Flexible Modeling Choices, and Multiple-Testing Burden

The modern statistical consensus in psychiatric genetics is that candidate–gene main effects and interactions reported in small to moderate samples have often failed to replicate at scale. Large evaluations across multiple cohorts find little support for many historical depression candidate genes and candidate G × E claims, and methodological reviews have detailed how publication bias, underpowered interaction tests, population stratification, and analytic flexibility can generate apparently “large” interaction effects that shrink toward zero in better-powered designs [50,51].

This does not mean that every moderation signal is false—rather, it means that the distribution of published interaction effects is expected to be wide, with occasional striking estimates that are partly “winner’s curse,” especially when subgrouping (e.g., by sex, adversity, treatment arm) reduces effective N. Practitioner-focused critiques of G × E also emphasize that, even when interactions are real, they tend to be small and context-specific, requiring careful preregistration, harmonized measures, and replication [52].

#### 4.1.5. The Genetic Architecture of Depression Is Highly Polygenic—So Single Variants Rarely Explain Consistent Moderation by Themselves

Major depression risk is spread across many variants of tiny effect, with extensive pleiotropy. Reviews of GWAS methods and genetic architecture emphasize that complex traits are typically highly polygenic; this makes it statistically unlikely that one functional polymorphism will deliver robust, generalizable moderation across ages, populations, and intervention types [53,54].

This polygenic reality helps reconcile why polygenic risk score approaches often show more stable “main effect + lifestyle effect” patterns than candidate-gene moderation: the genetic signal is aggregated, reducing idiosyncratic noise from any single locus. It also clarifies why two studies can both be “right” in different ways: a candidate variant may influence responsivity in a narrow developmental or stress-exposure window, while broad population-level protection from physical activity is better captured by polygenic liability plus behavior measured well.

#### 4.1.6. Developmental Stage and Sex Are Not “Nuisance Variables”—They Change Neurobiology, Stress Sensitivity, and the Meaning of Activity Exposure

Adolescence is a period of heightened neuroplasticity, changing gonadal hormones, and evolving stress reactivity. In that context, a plasticity-related variant could plausibly interact with activity or stress because the underlying systems (e.g., BDNF-dependent synaptic remodeling, HPA calibration) are themselves in flux. By later adulthood, variance in depressive symptoms may be driven more by medical comorbidity, inflammation, disability, and social loss—shifting which exercise mechanisms dominate and which genetic differences matter. Evidence that Val66Met relates to hippocampal or amygdala-linked processing, and that early-life stress can confound or modify these relationships, supports the plausibility of age- and context-specific effects rather than a universal interaction [45,55].

#### 4.1.7. “Physical Activity” Is Not One Exposure: Dose, Intensity, Pattern, and Adverse Responses (e.g., Overreaching/Overtraining) Matter

Some included results suggest that very high activity can associate with more symptoms in unusually active samples. That is not necessarily contradictory to an overall protective effect—because extreme training loads can produce fatigue, sleep disruption, immune perturbations, and mood symptoms in susceptible individuals (often discussed under overreaching/overtraining syndromes). The broader exercise–physiology literature recognizes that mental-health responses can be U-shaped in certain contexts: moderate, sustainable activity is typically beneficial, while excessive, poorly recovered training can be destabilizing—again creating conditions where moderation appears in one sample and not another.

Putting it together mechanistically: the reviewed pattern is most consistent with a model in which physical activity has multiple partially independent antidepressant routes (plasticity/BDNF-related synaptic remodeling, inflammation and myokine signaling, metabolic/kynurenine detoxification, circadian and sleep effects, reward/monoamine changes), and genetic variation can influence (a) baseline vulnerability in these systems, (b) the gain of the system’s response to exercise, and (c) the probability of engaging in/maintaining exercise. When depression phenotypes, exposure measurement, and context (stress, sex, age, comorbidity, training dose) align with a pathway where a given variant matters, moderation can look “strong”. When they do not—or when sample size and measurement error dominate—results tend toward null. The field-wide lesson from psychiatric G × E research is that this variability is not a nuisance to explain away; it is a predictable consequence of small interaction effects layered on heterogeneous biology and heterogeneous measurement, and it is exactly why larger, preregistered, well-measured (ideally device-based) studies—and polygenic rather than single-variant models—tend to yield more reproducible patterns.

### 4.2. Mechanistic Pathways That Plausibly Link Activity to Depression Risk and Response

Across the included studies, physical activity sometimes looks like a broadly protective “main effect” (e.g., lower incidence or symptom burden regardless of genetic risk), sometimes like a moderator (benefit concentrated in specific genotypes/sexes or specific symptom dimensions), and sometimes null. A useful way to integrate these mixed patterns is to treat “activity → depression” not as one pathway but as a bundle of partially independent biological and psychosocial channels whose relative importance changes with age, baseline symptom biology, treatment context, and the way activity is measured.

A central biological account is the neurotrophic/neuroplasticity pathway, in which depression is associated with reduced neurotrophic support and stress-related synaptic/structural remodeling, especially in hippocampal–prefrontal circuits, and effective interventions reverse parts of that deficit. The neurotrophic hypothesis and related work place BDNF signaling (and downstream synaptic plasticity, dendritic remodeling, and neurogenesis) near the center of depression vulnerability and recovery [56,57,58,59]. Physical activity is one of the most robust behavioral “inputs” to this plasticity system: in animals, voluntary running increases hippocampal neurogenesis and plasticity-related learning measures, and in humans, aerobic training can increase hippocampal volume and improve memory in late life—changes frequently interpreted as evidence that activity engages brain-maintenance and plasticity processes relevant to mood regulation [60,61]. At the peripheral level, many studies report increases in circulating BDNF after acute and/or chronic exercise (with substantial heterogeneity by assay, timing, platelet effects, and population), and mechanistic reviews emphasize that “BDNF-related” effects are plausible yet not reducible to a single serum biomarker pattern [14,62,63]. This helps interpret why some included exercise–BDNF genotype interactions appear clinically meaningful while serum BDNF change often fails to track symptom response: circulating BDNF is an imperfect proxy for activity-dependent signaling in specific brain circuits, and “plasticity” can be realized through multiple downstream routes even when serum BDNF is noisy.

A second major channel is immune–inflammatory and oxidative-stress regulation. A large literature supports that subsets of depression—particularly with prominent somatic symptoms, anhedonia, or treatment resistance—are associated with chronic low-grade inflammation and altered immune–brain signaling that can influence neurotransmission and reward/stress circuits [8,64,65]. Exercise can downshift inflammatory tone and improve redox balance via repeated anti-inflammatory “pulses” (myokine signaling, improved metabolic regulation, and autonomic rebalancing), which may matter most when baseline inflammation is elevated. Mechanistically oriented syntheses of exercise in depression often highlight that these immune effects can converge on monoamine systems, glutamatergic function, and microglial activation states, providing plausible explanations for why activity may preferentially improve somatic symptom clusters in some cohorts (fatigue/sleep/appetite-related complaints are strongly linked to inflammatory signaling) even when total depression scores change modestly [14,64]. In other words, “exercise works” in part by changing the body–brain interface that shapes sickness-behavior-like symptom dimensions; the signal is easiest to detect when those processes are actually driving symptoms.

A closely related and increasingly specific mechanism is the tryptophan–kynurenine pathway. Stress and inflammation can push tryptophan metabolism toward kynurenine and downstream neuroactive metabolites, some of which are neurotoxic or excitotoxic and have been implicated in depressive-like phenotypes. Exercise training induces skeletal-muscle programs (notably PGC-1α1-related) that increase kynurenine aminotransferase activity and shift kynurenine toward kynurenic acid, reducing the pool of circulating kynurenine available to enter the brain—an elegant muscle-to-brain resilience pathway demonstrated in preclinical work [9]. This framework also clarifies why objective activity measures sometimes show stronger causal signals than self-report in genetic studies: if the relevant biology is dose-, intensity-, and consistency-dependent (and intertwined with inflammatory load), measurement noise in self-report can wash out the part of exposure that truly engages these metabolic shifts.

Beyond neurotrophins and immunometabolism, activity also affects depression through stress-system and autonomic regulation. Many depressed patients show altered HPA-axis dynamics (not always “high cortisol,” but dysregulated stress reactivity, feedback sensitivity, and diurnal rhythms). Activity can function as repeated, controllable “stress inoculation”, improving parasympathetic tone and stress recovery, and—in some samples—reducing cortisol or normalizing stress physiology, particularly when baseline dysregulation is present [66,67]. Importantly, these effects are not monotonic: very high training loads, poor recovery, or overtraining-like states may increase fatigue, sleep disruption, and mood symptoms, which provides a biologically coherent basis for the occasional “high activity looks worse” findings in highly active convenience samples (where “high” may mean extreme). This is consistent with broader physiological models in which the mental-health impact depends on the balance between adaptive conditioning and allostatic overload.

Several muscle-derived signaling molecules (“exerkines”) provide additional mechanistic bridges between activity and mood-related brain function. For instance, cathepsin B has been proposed as an exercise-induced factor linked to hippocampal adaptations and cognition; human and animal evidence suggests it rises with exercise and can participate in neurogenic/plasticity cascades, though translation to mood endpoints and biomarker standardization remain open questions [68,69]. Irisin/FNDC5 is another candidate pathway: exercise-induced peripheral signals may influence brain BDNF-related programs and stress responses, and emerging clinical/translation work connects altered FNDC5/irisin-related biology with depression, albeit with mixed results across tissues and paradigms [70,71]. These exerkine frameworks make sense of moderation patterns in genetic studies: if the “active ingredient” differs across people (e.g., inflammatory downshifting vs. neurotrophic plasticity vs. metabolic rerouting), then the same minutes/week can produce different biological consequences depending on genotype, sex hormones, age-related muscle/immune changes, and comorbidity.

Activity also engages reward, affect, and neuromodulator systems that are core to depressive symptomatology. Acute and chronic exercise can influence monoaminergic signaling (serotonin, dopamine, norepinephrine), motivational salience, and reward learning—functions that are impaired in anhedonic depression. While the neurotransmitter story is complex and not reducible to a single “serotonin increases” claim, it is biologically plausible that individuals whose vulnerability is more serotonergic–stress–reactivity-linked (or whose treatments target these systems) would show different response profiles to activity, consistent with candidate-gene-by-activity interactions observed in some small studies and intervention contexts. At the same time, modern mechanistic thinking emphasizes that reward improvements can be mediated by behavioral activation and reinforcement learning mechanisms as much as by neurotransmitter concentrations per se—again, multiple routes to the same clinical endpoint [14].

A particularly well-supported affective mechanism is the endocannabinoid system. Human endurance exercise increases circulating endocannabinoids, and animal work demonstrates that cannabinoid receptor signaling is necessary for key “runner’s high” features (anxiolysis/analgesia and affective shifts), supporting a plausible pathway from sustained rhythmic activity to acute mood improvement and longer-term reinforcement of activity as a self-maintaining behavior [72,73]. This is relevant to depression both because anxiolysis and stress-buffering reduce risk trajectories, and because positive affect during/after activity increases adherence—turning a one-off exposure into a sustained habit capable of driving neuroplastic, metabolic, and inflammatory adaptation.

Another broad conduit is the gut–brain axis. Exercise can alter gut microbiota composition and metabolite profiles, which in turn influence immune tone, vagal signaling, tryptophan/indole metabolites, and neuroactive compound production; mechanistic reviews increasingly integrate gut-mediated immune and metabolic routes as contributors to exercise’s antidepressant effects [74,75]. This pathway is especially consistent with findings where activity effects vary with lifestyle context (diet, adiposity, sleep, comorbidity), because microbiome-related effects are strongly context-dependent.

Finally, the mechanistic story is incomplete without psychosocial and behavioral pathways, which often interact with biology rather than competing with it. Physical activity can increase social contact (especially in group formats), self-efficacy, perceived control, and daily structure; it can also reduce rumination by shifting attention and providing mastery experiences. These mechanisms align with behavioral activation models of depression and can be particularly relevant to prevention (incident depression) and to maintenance of gains. Contemporary syntheses argue that the strongest and most reproducible effects of activity on depression likely reflect convergence: modest changes across plasticity, immune-metabolic regulation, stress physiology, sleep/circadian timing, and reinforcement processes that together shift risk and symptom trajectories [14].

Putting these pathways together helps explain why the included genetic-interplay evidence is not uniform. Different studies emphasize different activity “doses” (light vs. vigorous, total volume vs. timing, leisure-time vs. structured training), different depression phenotypes (incident diagnosis vs. symptom dimensions vs. short-term treatment response), and different biological bottlenecks (plasticity-limited vs. inflammation-driven vs. stress-reactivity-dominant depression). If activity’s antidepressant impact is multicomponent, then moderation signals will appear when a study’s exposure/outcome choices happen to line up with the dominant pathway in that sample—and will fade when they do not.

### 4.3. Interpreting Candidate–Gene Moderation in the Modern Genetics Era

The candidate–gene moderation signals in this review sit at an awkward but scientifically productive intersection: they are often biologically plausible (e.g., neurotrophin signaling, serotonergic regulation, stress biology), yet they appear against a background in which modern psychiatric genetics has repeatedly shown that major depression is highly polygenic, with risk distributed across many loci of very small marginal effect. Large GWAS meta-analyses have now identified dozens to hundreds of associated loci, and the emerging picture is not one of a few “key” common variants exerting sizable effects, but rather a diffuse architecture involving synaptic, neuronal, and regulatory pathways spread across the genome [2,76,77]. This broad polygenicity shifts the prior probability for strong, reproducible moderation by any single common candidate polymorphism: it is not impossible, but—absent unusually strong functional evidence and very large, well-measured samples—it is statistically unlikely that one SNP will reliably deliver a large and portable interaction with a complex, variably measured exposure like physical activity.

This matters because candidate–gene interaction studies historically tended to report interaction effects that were large relative to what polygenic models would predict, frequently in modest samples with flexible analytic choices (multiple outcomes, multiple environmental definitions, subgroup analyses by sex/age/treatment arm, alternative genotype coding, different covariate sets). The field has learned—painfully—that such conditions create fertile ground for false positives, even when investigators act in good faith. The general statistical logic is well captured by the “low prior + multiple testing + modest power” framework: when many hypotheses are implicitly tested, the proportion of significant findings that are true can be low even at conventional α levels [78]. In psychiatric genetics specifically, critical reviews have emphasized that candidate G × E work often lacked the sample sizes and measurement precision needed for stable inference, and that analytic flexibility can produce apparently compelling interactions that fail to replicate [51,79].

A particularly influential modern check on the candidate-gene era is the large-sample reassessment showing no support for historical depression candidate genes or candidate-gene-by-interaction hypotheses when tested at scale, suggesting that many earlier “hits” were likely false positives rather than simply underpowered truths waiting to be confirmed [50]. Importantly, this does not imply that the biological pathways (BDNF signaling, serotonin transport, HPA-axis reactivity) are irrelevant to depression or to exercise response. It implies something narrower and more actionable: common, single candidate variants chosen a priori rarely serve as reliable, stand-alone proxies for pathway-level susceptibility in depression.

#### 4.3.1. Why “Plausible Biology” Did Not Guarantee Replicable Moderation

Candidate variants were often selected because they were thought to be functional. However, “functional” in a molecular assay does not guarantee that a polymorphism is a good predictor of clinically meaningful variation in a heterogeneous phenotype like depression—especially when (i) the effect of the variant on the protein/pathway is modest or context-dependent, (ii) compensatory mechanisms exist, and (iii) the phenotype aggregates multiple causal routes. Even more concretely, some widely used candidate polymorphisms are not cleanly functional in the way early studies assumed, and mismeasurement at the genotype level can erase true effects or create artifacts.

A classic example is 5-HTTLPR. Much candidate work treated it as biallelic (S vs. L), but functional data indicate a triallelic situation because the L allele can be subdivided (e.g., via rs25531) into forms with different transcriptional activity; collapsing these together introduces genotype misclassification that inflates noise and can distort interaction patterns [80,81]. This is not a pedantic detail: when an exposure (activity) and outcome (depression symptoms/diagnosis) are also measured with error, the interaction term becomes especially unstable—sometimes attenuated toward null, sometimes spuriously significant depending on the joint structure of measurement error and covariate imbalance.

#### 4.3.2. Confounding and “Interaction-Specific” Adjustment Problems

A second modern lesson is that even when main effects are properly adjusted, interaction terms can remain confounded unless covariates are modeled in an interaction-aware way. In G × E settings, standard practice of adjusting for covariate main effects can be insufficient: if a covariate is related to the environment and to genotype (directly or via ancestry), then failing to include covariate × genotype and covariate × environment terms can leave residual structure that masquerades as G × E. This issue is especially relevant for physical activity because activity is strongly patterned by socioeconomic position, health status, and social context—factors that can correlate with genetic ancestry and with depression risk. Keller’s methodological critique highlights how pervasive “incomplete” confounder control has been in the G × E literature and why this can produce non-replicable interactions [82].

Relatedly, population stratification is not just a main-effect problem: it can be an interaction problem. If allele frequencies differ by ancestry and activity patterns also differ by ancestry-linked social environments, spurious moderation can appear. Candidate–gene studies were often conducted in convenience samples where ancestry was not well characterized or adequately controlled, increasing this risk.

#### 4.3.3. Publication Bias, Small-Study Effects, and the “Winner’s Curse” for Interactions

Interaction effects are typically smaller and harder to detect than main effects, so underpowered studies are particularly vulnerable to exaggerated estimates in the subset of analyses that reach significance (a small-study “winner’s curse” dynamic). This helps explain why the literature can show a spectrum from strong moderation signals to nulls even when the underlying truth is modest. Methodological reflections and recommendations for the field have stressed preregistration, standardized measurement, careful multiple-testing control, and—most importantly—large samples with replication as the default standard for candidate G × E claims [79,83]. The general warning about selective significance and low positive predictive value in “search-heavy” literatures applies here with full force.

#### 4.3.4. What the 5-HTTLPR Story Teaches (And What It Does Not)

The serotonin-transporter-by-stress hypothesis is historically important because it illustrates how an intuitively coherent mechanism can generate decades of mixed empirical results. The original high-profile report suggested that genotype moderated the impact of stress on depression, sparking extensive follow-up [84]. Yet large meta-analyses have often not supported a robust interaction once broader evidence and harmonized analyses are brought to bear [85]. (Where individual studies can still find moderation under specific designs and measures, meta-analytic synthesis has tended to reduce confidence that the interaction is general and strong).

The implication for gene–physical activity moderation is not that moderation is impossible, but that candidate–gene moderation should be treated as hypothesis-generating unless supported by: (i) large, well-powered samples, (ii) objective or well-validated exposure measurement, (iii) rigorous interaction-aware confounder control, (iv) ancestry control, and (v) pre-specified models with successful replication. When these conditions are absent, the posterior probability of a “true, portable” candidate-gene moderation effect is low.

#### 4.3.5. Moving from Candidate Genes to Polygenic Moderation and Causal Triangulation

Modern genetics provides two upgrades that are directly relevant for interpreting the mixed candidate findings in this review.

(a) Polygenic risk and pathway-distributed susceptibility. Instead of treating a single SNP as “the” moderator, polygenic risk scores (PRS) aggregate thousands of variants, better matching the known architecture of depression. Large GWAS and reviews emphasize that PRS capture meaningful but still modest fractions of variance, and that interpretation requires careful attention to ancestry portability, phenotype definition, and reporting standards [2,78]. PRS frameworks also naturally align with the idea that physical activity may shift risk across the spectrum of underlying liability rather than only in one genotype-defined subgroup. This does not guarantee large PRS × activity interactions—those too may be small—but it greatly improves biological and statistical plausibility compared with single-candidate moderation.

(b) Triangulation with causal inference tools. Mendelian randomization and within-family designs do not “solve” mechanisms, but they help adjudicate whether an activity–depression association is plausibly causal versus driven by shared genetics or confounding. The broader depression genetics literature increasingly argues for converging evidence across GWAS/PRS, MR, randomized trials, and family-based designs because any one approach can be biased in predictable ways [2,76].

### 4.4. Polygenic Susceptibility, Mendelian Randomization, and What Looks Most Robust

Across the included literature, the polygenic and Mendelian randomization (MR) strands converge on a comparatively consistent picture: (i) genetic liability to depression is highly polygenic and diffuse, (ii) physical activity shows a broadly protective association that often persists across polygenic-risk strata, and (iii) the most “robust-looking” causal evidence tends to appear when physical activity is objectively measured (or when inactivity is instrumented strongly), while results are weaker and more heterogeneous for self-reported activity. This triangulation matters because conventional observational designs remain vulnerable to confounding and reverse causation, while candidate–gene moderation faces well-known replicability constraints.

#### 4.4.1. Polygenic Susceptibility: What PRS Can and Cannot Tell Us

Modern depression genetics shows that liability is spread across many loci of very small effect, consistent with large-scale GWAS that identified dozens to hundreds of associated variants and emphasized extensive polygenicity rather than a few large “depression genes” [3,39]. This architecture implies that (a) any single environmental exposure (including physical activity) is unlikely to interact strongly with one variant in a stable way, and (b) polygenic risk scores (PRS) are a more coherent way to summarize inherited susceptibility—yet they remain probabilistic and context-dependent. Methodologically, PRS construction has evolved rapidly (e.g., Bayesian shrinkage approaches such as PRS-CS), improving signal extraction from GWAS summary statistics but not eliminating the fundamental small-effect reality [86]. In parallel, the field has moved toward reproducibility and transparency (e.g., PRS reporting standards and public repositories such as the PGS Catalog), reflecting awareness that PRS performance depends on choices about discovery GWAS, clumping/shrinkage, ancestry matching, and phenotype definition [87,88,89].

Within this framework, the most credible interpretation of PRS–lifestyle results are additive and partially independent contributions: PRS captures stable liability, while behavior captures modifiable exposure (plus correlated social/health context). That pattern aligns with broader epidemiologic evidence that physical activity is associated with lower incident depression risk across cohorts and dose ranges, often with diminishing returns at higher levels [90]. The practical implication is not that PRS identifies who “will” become depressed, but that higher PRS shifts baseline risk upward, and activity may shift it downward—often without requiring a gene-specific “match.” This is exactly the scenario where PRS is most useful: stratification and calibration in large samples, not deterministic individual prediction.

Two caveats are central when interpreting “activity offsets PRS risk” claims. First, PRS portability across ancestries remains limited because discovery GWAS are still disproportionately European-ancestry; applying a European-derived PRS to other populations can degrade predictive validity and potentially widen disparities [91,92]. Second, PRS–behavior associations can reflect gene–environment correlation (genetic influences on activity propensity, socioeconomic attainment, health status, etc.), so “PRS is independent of activity” should be evaluated rather than assumed—especially when activity is self-reported and socially patterned [90,93].

#### 4.4.2. Why MR Strengthens the Inference—And Where It Still Strains

MR is often framed as using genetic variants as “anchors” for causal inference, because alleles are randomly assorted at conception and therefore less confounded by later-life social/behavioral factors [93,94]. In practice, MR credibility depends on instrument strength and validity (no horizontal pleiotropy, correct directionality, etc.), and modern MR studies increasingly follow formal reporting guidance and sensitivity workflows (e.g., STROBE-MR; pleiotropy diagnostics such as MR-PRESSO) [95,96]. Against that backdrop, the repeated observation that accelerometer-based activity instruments yield clearer protective estimates for depression than self-report instruments is not surprising: self-report activity is noisier, more biased, and more entangled with mood, self-perception, and health limitations—features that weaken instruments and inflate heterogeneity. This measurement issue is also visible outside MR: device-based cohort analyses tend to show cleaner dose-response shapes than self-report [90,97].

At the same time, MR results also highlight genuine bidirectionality: genetic liability to depression can reduce activity, consistent with motivational, fatigue, and anhedonia pathways that make physical activity harder to initiate or sustain. The presence of this reverse pathway means that even strong prospective cohort associations can partially reflect “depression → inactivity” rather than “inactivity → depression.” MR is valuable here precisely because it can test directionality, but reverse-direction MR can also be underpowered or distorted if the activity instrument is weak [93,95].

#### 4.4.3. What Seems Most Robust Across Designs

When placing PRS stratification, cohort analyses, intervention syntheses, and MR side-by-side, several conclusions look sturdier than others:

(a) Being physically active is consistently associated with lower depression risk, including at “below guideline” doses in large meta-analytic syntheses, suggesting that the threshold for benefit is not extremely high [90].

(b) Exercise interventions reduce depressive symptoms on average, with effect sizes that vary by clinical status, comparator, and study quality, but the overall direction is stable across major reviews and meta-analyses [98,99]. This is compatible with a causal effect, though it does not establish which biological pathways mediate benefit or which individuals benefit most.

(c) Objective activity measures produce more coherent causal signals in genetic analyses than self-report measures, supporting the idea that measurement error and reporting bias are major reasons why some genetic findings look inconsistent across studies [95].

(d) PRS can identify baseline liability gradients, but lifestyle associations often appear broadly similar across PRS strata, implying that physical activity is not merely helpful for “low-risk” individuals. This is consistent with a model where activity shifts risk distribution in the population rather than “turning off” polygenic risk [89,90].

(e) Generalizability is still constrained, especially by ancestry (PRS portability), phenotype heterogeneity (clinical diagnosis vs. symptom scale vs. EHR codes), and exposure heterogeneity (domain, intensity, timing, and measurement method). The polygenic era has improved statistical power, but it has also clarified that “one PRS” or “one activity metric” rarely captures the full etiologic complexity [87,92].

### 4.5. Dose, Intensity, Timing, and “When/What Kind” of Activity Might Matter

Across the studies in this review, “physical activity” is not a single exposure: it varies by dose (volume), intensity, patterning (how activity is distributed across days), timing (time of day), and type (walking vs. resistance training vs. mind–body, etc.). Heterogeneity across these dimensions is a major reason why some analyses detect strong signals (including gene-by-activity moderation) while others return null results—even when all are plausibly studying the same broad phenomenon.

#### 4.5.1. Dose: A Steep Early Benefit Curve and Likely Plateaus (And Occasional U-Shapes)

Large-scale observational syntheses consistently suggest an “L-shaped” dose–response curve: the biggest marginal mental-health gains occur when moving from none to some activity, with diminishing returns at higher volumes. In a pooled analysis focused on depression prevention, modest volumes of activity were associated with meaningfully lower incident depression risk, and higher volumes added smaller incremental benefit [5]. Similar “most benefit at low-to-moderate doses” patterns appear in population-based dose–response work that explicitly models nonlinearity, which matters because studies sampling very active groups (e.g., sports science students, endurance cohorts) can end up testing the flat part of the curve, making true effects harder to detect and potentially producing unusual interaction patterns [100,101].

Daily step-count evidence illustrates the same principle using an intuitive metric. A systematic review/meta-analysis of >90,000 adults found higher objectively measured steps associated with fewer depressive symptoms, with notable benefits already around ~5000–7000 steps/day and smaller marginal gains beyond that range [102]. Importantly, step counts also capture “incidental” movement (non-exercise activity thermogenesis), which may partly explain why step-based associations sometimes look stronger than associations for narrowly defined leisure-time exercise questionnaires.

At the other end of the dose range, some datasets and reviews suggest that very high training loads can be associated with worse mood in some individuals (often discussed under overreaching/overtraining frameworks). This is conceptually relevant to moderation results like the “high activity” reversal reported in highly active student samples: if the comparison is not “inactive vs. active” but “active vs. extremely active,” the biological and psychological correlates may change (fatigue, sleep disruption, injury, compulsive exercise) [100,103]. The practical implication is that gene-by-activity effects may differ at low-to-moderate doses (where activity is largely restorative) versus very high doses (where stress physiology and recovery become limiting factors).

#### 4.5.2. Intensity: Light-to-Moderate Activity Is Often Sufficient; Vigorous and HIIT Are Not Uniformly Superior

Across epidemiologic studies, moderate-to-vigorous physical activity (MVPA) often predicts lower depression risk, but not always more strongly than lighter activity once confounding and measurement error are considered. Some large cohorts report particularly consistent associations for light activity or mixed-intensity movement—possibly because light activity is easier to sustain, better tolerated during low mood, and more tightly coupled to social exposure and time outdoors (all relevant to depression) [99,102].

In intervention research, meta-analytic evidence supports antidepressant effects across multiple exercise modalities and intensities, but average effects can be attenuated by floor effects (low baseline symptoms), adherence differences, and heterogeneity in comparators. A major umbrella-style synthesis in BMJ (2024) reported clinically meaningful symptom reductions for several exercise forms, with effects depending on intervention characteristics and populations studied [104]. For resistance training specifically, meta-analytic evidence supports reductions in depressive symptoms across diverse adult samples, indicating that “type” and intensity can be varied while still producing benefit [105]. For HIIT, systematic reviews generally find improvements in depression symptoms in some contexts, but effects are less consistently demonstrated across clinical groups and may be more sensitive to adherence, tolerability, and baseline severity than moderate continuous training [103].

This matters for gene–activity interplay because intensity changes the mix of biological signals (lactate, catecholamines, inflammatory responses, thermoregulation), psychological experiences (mastery vs. threat), and adherence barriers. If a genotype moderates stress reactivity or neurotrophin signaling, its interaction might be more visible at intensities that strongly perturb those pathways—yet those same intensities can increase dropout and measurement noise, which pushes results toward null.

#### 4.5.3. Patterning: “Weekend Warrior” vs. Regular—Total Volume May Matter More than Frequency

A key modern finding from accelerometer-based cohorts is that meeting a weekly MVPA threshold is associated with lower subsequent depression/anxiety risk whether the activity is spread across the week or concentrated into 1–2 days (“weekend warrior”). Earlier population work on psychological distress also pointed in the same direction: compared with inactivity, weekend-warrior patterns were associated with lower odds of common mental disorder, suggesting that frequency is not necessarily the active ingredient once dose is held constant. This helps reconcile why some studies focusing on “sessions per week” find weak or inconsistent results—session frequency is a noisy proxy for total energy expenditure, and it can misclassify individuals who accumulate activity in longer bouts.

Mechanistically, this aligns with two partially separable pathways: (i) acute mood and anxiety effects (often stronger right after exercise and potentially sensitive to frequency), and (ii) chronic adaptations (cardiorespiratory fitness, sleep consolidation, inflammatory tone) that depend more on cumulative training load. If depression outcomes are assessed months or years later, cumulative load and fitness are more likely to dominate.

#### 4.5.4. Timing (“When”) and Circadian Alignment: Emerging Evidence That Earlier Activity May Be Favorable

The newest accelerometer-based prospective evidence suggests that when activity occurs may matter for depression risk, independent of total volume. In UK Biobank–scale analyses, earlier timing profiles (e.g., early-morning peaks in total activity, or morning/midday MVPA) have been associated with lower incident depression risk compared with later timing profiles, even after adjusting for total activity volume. Although not all timing studies agree and residual confounding remains possible (employment schedules, caregiving, baseline sleep/circadian problems), the signal is plausible given known circadian biology.

Exercise is a zeitgeber: it can shift circadian phase and strengthen circadian amplitude, with effects that depend on time of day and chronotype, and these circadian changes intersect with depression biology (sleep architecture, cortisol rhythms, monoaminergic signaling, reward sensitivity). Morning activity may also co-occur with bright light exposure and earlier sleep timing, both linked to better mood regulation in many (not all) individuals. A reasonable integrative hypothesis is that earlier activity is beneficial when it improves circadian alignment and sleep continuity, while late-evening vigorous activity could be neutral or adverse for those prone to insomnia or delayed phase—conditions tightly coupled to depression onset and persistence.

#### 4.5.5. Type (“What Kind”): Walking, Resistance Training, and Mind–Body Exercise May Operate Through Partially Distinct Routes

Several MR and large-cohort analyses increasingly suggest that not all “activity types” are equivalent in their relationship to depression. For example, some genetic-instrument analyses report protective associations for accessible activity forms like walking and “other/aerobic exercise,” and harmful associations for inactivity, though effect size estimates vary and are sensitive to phenotype definition and instrument strength. Observational step-count meta-analysis similarly supports walking/steps as a meaningful, scalable behavioral target. Resistance training has separate supportive evidence and may be particularly relevant for individuals with somatic symptoms, fatigue, or metabolic comorbidities, via functional gains and inflammation/insulin pathways in addition to classic “endorphin/monoamine” narratives. Mind–body activities (e.g., yoga, tai chi) show antidepressant effects in some meta-analyses and may be especially potent where stress physiology and interoceptive regulation are central, though heterogeneity in trial quality and comparators is substantial.

#### 4.5.6. Why These Dimensions Matter for Interpreting Gene–Activity Findings in This Review

Candidate–gene moderation signals are most likely to appear when (a) the exposure is measured with enough resolution to capture biologically meaningful contrasts (not just “active vs. inactive”), and (b) the exposure range spans the steep part of the dose–response curve. Timing- and pattern-based accelerometry studies meet (a) by quantifying when and how activity occurs; clinical trials meet (b) if they enroll sedentary or symptomatic participants with room to improve; but many cross-sectional volunteer samples meet neither (restricted range, high baseline activity, low symptoms). The net result is exactly the spread seen here: strong signals in some exposure definitions (e.g., objectively measured activity, clinically meaningful baseline risk, or timing-aware phenotypes) and nulls where exposure misclassification, restricted range, or competing mechanisms (overtraining, pain, sleep disruption) wash out average effects.

## 5. Limitations and Future Directions

### 5.1. Key Limitations in the Current Evidence Base

#### 5.1.1. Low Prior Plausibility and Weak Reproducibility in Candidate–Gene and Candidate × Environment Designs

A substantial portion of the older literature on gene–environment interplay in psychiatry was built around a small set of “biologically plausible” candidate variants, often tested in modest samples with many analytic degrees of freedom (multiple outcomes, alternative genotype codings, subgroup analyses). This combination inflates false-positive risk and undermines replicability, even when conventional p thresholds are used. Methodological reviews of the first decade of candidate G × E research document patterns consistent with publication bias, underpowered interaction tests, and selective reporting. Large-scale evaluations of historical depression candidate–gene claims further suggest that many earlier “hits” are unlikely to generalize.

#### 5.1.2. Chronic Underpowering for Interaction Effects and Unstable Estimates

Gene × physical activity (G × PA) interaction effects—especially at single-variant resolution—are typically small. Detecting them reliably requires far larger samples than are needed for main effects, plus careful control of multiple testing and model specification. When interaction analyses are run in small-to-moderate samples, effect sizes are prone to winner’s curse and can flip direction across cohorts. These problems are well recognized across behavioral and biomedical sciences when studies are low-powered and analytic flexibility is high.

#### 5.1.3. Measurement Limitations: Physical Activity Is Often Misclassified, and Misclassification Is Not Random

Many studies rely on brief self-report activity questionnaires, which correlate only modestly with objective measures and can introduce systematic error (e.g., recall bias, social desirability, ceiling/floor effects). Systematic reviews comparing self-report to direct measurement show limited agreement, with error structures that can bias associations and interactions in unpredictable ways. Because interaction tests are particularly sensitive to measurement error in either the moderator (PA) or outcome, even “small” misclassification can wash out true effects—or generate spurious ones—unless objective monitoring, repeated assessments, and calibration substudies are incorporated.

#### 5.1.4. Depression Phenotyping Is Heterogeneous and Often Treated as a Single Score

Depression is not a unitary construct: different symptom profiles can yield the same sum-score, and genetic/environmental influences may map more strongly onto particular symptom dimensions (e.g., anhedonia vs. somatic symptoms) than onto a total score. This matters because G × PA effects could be symptom-specific; collapsing across heterogeneous manifestations can dilute signal and produce inconsistent findings across cohorts that use different instruments, time windows, or diagnostic definitions.

#### 5.1.5. Confounding, Gene–Environment Correlation, and Selection Processes Remain Difficult to Rule Out

Physical activity is socially patterned and correlates with health status, personality, socioeconomic resources, and comorbidity—factors that also relate to depression risk. Even strong covariate adjustment can leave residual confounding. In addition, gene–environment correlation (rGE)—where genetic propensities influence exposure to environments/behaviors such as activity—can mimic or distort interaction patterns if not explicitly modeled (e.g., via within-family designs, negative controls, or longitudinal frameworks that separate selection into PA from PA effects).

#### 5.1.6. Limited Ancestral Diversity and Poor Portability of Polygenic Indices

Much of the genomics informing depression PRS and many G × E analyses is derived from European-ancestry GWAS. As a result, PRS performance drops in other ancestry groups, and effect estimates can be biased by population structure, differences in linkage disequilibrium, and exposure distributions. This limitation constrains generalizability and raises equity concerns if results are translated into stratified prevention or “precision lifestyle” recommendations.

#### 5.1.7. Mendelian Randomization Adds Causal Leverage, but Key Assumptions Are Nontrivial for Behavior and Mental Health

MR can reduce confounding and reverse causation, but it is vulnerable to weak instruments (common for behavioral phenotypes), horizontal pleiotropy, sample overlap, and phenotype–definition mismatch between exposure and outcome GWAS. These issues can be especially salient for physical activity because genetic instruments may capture propensity toward certain activity types or correlated traits (e.g., cardiometabolic factors) rather than modifiable behavior per se. Robust MR in this domain therefore requires transparent sensitivity analyses, triangulation across instruments/phenotypes, and careful interpretation of what the instrument actually represents.

#### 5.1.8. Fragmentation of Exposure Definitions and Limited Attention to Context

Across studies, “physical activity” can mean total volume, intensity, timing, frequency, structured exercise, leisure-time activity, occupational activity, or broad lifestyle movement—each with different biological and psychosocial correlates. When exposure definitions vary widely and context (sleep, sedentary time, medication, comorbidity, stress load) is inconsistently measured, it becomes difficult to reconcile results or to identify which component of “being active” plausibly interacts with genetic susceptibility. Methodologically, this motivates harmonized PA phenotypes, multivariable models that include correlated behaviors, and designs that separate acute exercise effects from long-term activity patterns.

### 5.2. What We Still Do Not Know

A major unresolved issue is what “depression” we are actually targeting when we talk about gene–physical activity interplay. Depression is not a single, uniform phenotype: the same total score (or diagnostic label) can reflect very different constellations of symptoms, trajectories, and underlying processes. Work on symptom-level heterogeneity shows that summing symptoms can obscure meaningful differences—both clinically and etiologically—and that the number of unique symptom presentations is extremely large. This matters for gene–behavior research because moderation effects could be present for some symptom dimensions (e.g., sleep/energy, anhedonia, psychomotor changes) but vanish when collapsed into a single scale. Until studies routinely model symptom profiles (and their stability over time) rather than only total scores or binary diagnoses, we will keep missing—or misattributing—where activity and genetic susceptibility truly intersect.

Closely related, we still do not know which parts of the physical-activity “exposure” are causal or biologically relevant. Physical activity is a composite of multiple dimensions—volume, intensity, frequency, bout patterning, modality, and context (leisure, occupational, transport), plus correlated behaviors such as sleep and sedentary time. Even with accelerometers, there is no single “best” phenotype: some metrics capture overall movement; others attempt to isolate MVPA; others describe within-day timing, rest–activity rhythm stability, and fragmentation. These features are not interchangeable, and different metrics can imply different mechanisms. The field has not yet converged on a principled measurement framework that specifies (a) which PA dimensions are hypothesized to matter for affective health and (b) which metrics most validly capture them, especially across age groups and clinical states. Work on rest–activity rhythm measurement highlights how device-derived circadian and fragmentation metrics can be biased by non-wear and processing choices, underscoring that even “objective” measures require careful validation.

A particularly open question is whether timing and regularity are independent levers or merely proxies. There is growing interest in when activity occurs (morning vs. later) and how regular daily rhythms are, but it is unclear whether these patterns represent causal exposures, consequences of underlying traits (chronotype, occupational constraints, caregiving), or confounded correlates of sleep timing and circadian misalignment. Reviews of chronotype and social jetlag emphasize that longitudinal pathway studies are still relatively scarce, and that disentangling mediation by circadian misalignment remains a key challenge. Without stronger causal designs that jointly model sleep timing, chronotype, and activity timing—ideally across repeated measurement waves—we cannot yet interpret “timing effects” as actionable targets rather than markers of broader lifestyle structure.

Another persistent unknown is how much of the mixed evidence is simply measurement error and construct mismatch. Self-report and device-based measures often diverge substantially, and the direction and magnitude of disagreement vary across tools and populations. Systematic reviews comparing direct (device) versus self-report PA show that self-report can both under- and over-estimate “true” activity, creating unpredictable bias and undermining cross-study comparability; the same concern applies to sedentary time measurement, where single-item self-reports can substantially underestimate sitting time relative to devices. These are not just nuisance issues: measurement error can attenuate associations, distort dose–response relationships, and make gene-by-behavior interactions appear inconsistent across studies that are, in reality, measuring different constructs.

We also still do not know where genetics primarily enters the pathway: risk, responsiveness, or adherence. Many genetic effects that look like “moderation of benefit” could instead reflect genetic influences on (i) selection into activity, (ii) the subjective affective response during activity, or (iii) maintenance and dropout over time. The affective response to exercise is a promising intermediate phenotype because it predicts future physical activity behavior in systematic evidence, suggesting a plausible bridge from biology to sustained behavior. If genetic variation shapes affective response (reward, stress reactivity, fatigue), then the long-run “protective” association of activity could be partly driven by genetically influenced adherence patterns rather than differential antidepressant responsiveness per unit of activity performed. That distinction matters for designing interventions: targeting enjoyment, perceived competence, and tolerability may be as important as prescribing dose.

A further gap is how to integrate PA with the full 24-h time-use composition. Time spent in MVPA, light activity, sedentary behavior, and sleep is co-dependent: increasing one necessarily displaces another. Analytic approaches using compositional data analysis were developed precisely to handle this constraint, yet they are not consistently adopted in gene–behavior work. Without compositional thinking, “activity effects” can be conflated with what activity replaces (sedentary time vs. sleep), making mechanistic interpretation and cross-study replication harder. The field still needs clearer standards for modeling substitution effects and for aligning genetic analyses with 24-h behavior compositions.

On causality, we still lack full clarity about how much confidence to place in genetic causal inference for behavioral exposures, and what those estimates mean for interventions. Mendelian randomization (MR) can strengthen causal interpretation, but PA traits often have relatively weak instruments, and results can be sensitive to pleiotropy and modeling choices. Contemporary MR guidance stresses the importance of checking instrument strength, exploring robust estimators, and understanding how weak instruments can bias estimates (often toward the null, but not always in more complex settings). For PA specifically, where genetic instruments may capture propensity or ability rather than behavior itself, translating MR estimates into “do X minutes of activity” prescriptions remains non-trivial. We still need clearer “estimate literacy” in this area: what exactly is being estimated, and how it maps (or fails to map) to modifiable exposures in trials.

A major unknown for any genetics-informed stratification is generalizability and fairness across populations. Most evidence—both for PRS and for gene-by-environment work—comes from European-ancestry datasets, and PRS portability across ancestries remains limited. Moreover, even within a single ancestry group, polygenic score performance can vary systematically by characteristics such as sex, age, and socioeconomic factors, complicating interpretation and raising concerns about bias if PRS are used to define “high-risk” groups. Until the field systematically validates PRS and interaction findings across diverse ancestries and social contexts—and improves methods for equitable prediction—precision recommendations based on genetic susceptibility remain premature.

Finally, we still do not know whether the overall picture converges when tested across methods chosen for complementary biases. Observational cohorts, RCTs, within-family designs, and MR each have characteristic weaknesses; no single design “solves” causality and moderation. A growing methodological consensus argues for deliberate triangulation—integrating evidence from approaches with different, ideally unrelated, bias structures—and being explicit about the expected direction of bias in each approach. For gene–physical activity interplay, that implies research programs designed prospectively to triangulate: harmonized phenotypes across cohorts, embedded randomization where possible, within-family analyses to reduce shared confounding, and genetic causal inference with transparent sensitivity checks. Without this kind of coordinated triangulation, it will remain difficult to distinguish true heterogeneity (real subgroup differences) from design-specific artefacts that merely look like heterogeneity.

### 5.3. Future Directions: How to Move the Field Forward

A first priority is to build an evidence base that is designed to detect small, context-dependent genetic effects without being dominated by false positives. That means moving away from underpowered, single-cohort candidate interaction analyses toward adequately powered, harmonized, multi-cohort efforts with prespecified hypotheses (or explicitly exploratory genome-wide scans) and transparent reporting. The broader psychiatric G × E literature has repeatedly shown how easily candidate interaction findings can arise from publication bias, analytic flexibility, and low power, and why replication in independent samples is non-negotiable if interaction claims are to be credible.

Second, future work needs better—and more comparable—measurement of physical activity and its correlates. Many observed inconsistencies across studies in behavioral genetics are plausibly explained by measurement error and construct mismatch (self-report capturing perceived activity and context, devices capturing movement volume and timing). A pragmatic path forward is to combine device-based measures (accelerometry, heart-rate wearables where available) with well-validated self-report that captures domains devices miss (occupational activity, caregiving load, perceived exertion, barriers, and motivation), and to harmonize processing pipelines across cohorts so “activity” means the same thing across datasets. Large-scale accelerometer resources and documentation (e.g., UK Biobank processing) show how feasible this is, and systematic comparisons highlight why device and self-report often relate differently to health outcomes.

Third, because time in a day is finite, activity research should more routinely adopt time-use and compositional approaches rather than treating activity dimensions as independent exposures. Modeling physical activity, sedentary behavior, and sleep as a composition respects their codependence and directly answers intervention-relevant questions (e.g., “what happens if 30 min of sedentary time is reallocated to light activity?”). This is especially important for gene–behavior interplay, because genetic liability may influence how time is redistributed (and with what correlates, such as sleep regularity) rather than simply shifting a single activity metric. Compositional data analysis frameworks and guidance in the movement–behavior literature provide well-developed tools that can be imported into the gene–environment interplay space.

Fourth, progress will depend on triangulating across complementary causal designs rather than treating any single design as decisive. Well-executed Mendelian randomization can strengthen causal claims, but it is sensitive to weak instruments, pleiotropy, and selection/overlap issues; accordingly, MR results should be routinely accompanied by modern reporting and robustness checks, and interpreted alongside other approaches that have different bias structures (longitudinal cohorts with repeated exposure/outcome measurement, within-family designs, quasi-experiments, and randomized trials where feasible). Triangulation, as formalized in epidemiology, is particularly well suited to the gene–activity–depression space because confounding and reverse causation are plausible in every observational analysis, and no single method fully resolves them.

Fifth, the field should more explicitly address genetic confounding pathways that can masquerade as “moderation.” For polygenic approaches, this includes ancestry-related portability limits (PRS often predicts less well outside the discovery ancestry, and even within ancestries prediction varies along continuous ancestry gradients) and family-level processes such as “genetic nurture”, where parental genotypes influence offspring environments and can bias naive interpretations of genotype–environment interplay. Incorporating within-family designs (siblings, parent–offspring trios) and reporting PRS construction/validation transparently will make interaction claims more interpretable and more generalizable. The emerging consensus in PRS methodology is to treat portability and family-level confounding as first-order design issues, not afterthoughts.

Sixth, future studies should minimize “researcher degrees of freedom” that are especially problematic for interaction tests (choice of outcome definition, exposure scaling, covariate sets, interaction functional form, subgrouping, genotype coding, and multiple-testing correction). Preregistration and analysis plans help separate prediction from post-diction, and specification-curve (multiverse) approaches can show whether an interaction is robust across reasonable analytic choices rather than being an artifact of one preferred specification. These practices are not cosmetic; they directly target the instability that has historically undermined confidence in complex, small-effect literatures.

Seventh, if the long-term goal is clinically useful personalization, the field needs a staged translation pathway that is realistic about effect sizes and ethical constraints. Early steps should focus on prediction of average benefit and risk with well-calibrated uncertainty, not deterministic genotype-based prescriptions. That implies reporting models in a way that supports calibration, external validation, and transportability testing, using established reporting frameworks for prediction tools and for polygenic scores. Only once robust, replicated effect modification is established should there be serious consideration of genotype-stratified or response-adaptive interventions—and even then, designs should prioritize equity (avoiding exclusion of underrepresented ancestries), feasibility, and safeguards against stigmatization.

Finally, mechanistic depth should be pursued in parallel with methodological rigor. Rather than adding isolated biomarkers opportunistically, future work can predefine mechanistic hypotheses that link (i) activity dose and pattern (including timing and composition), (ii) intermediate phenotypes (sleep regularity, inflammation, metabolic markers, neuroendocrine stress reactivity, neural plasticity markers), and (iii) depressive symptom dimensions. This “systems” framing fits naturally with triangulation: convergent evidence from repeated-measures cohorts, objective activity data, family-based genetic designs, and carefully reported MR can identify which pathways are most plausibly causal and for whom. The practical outcome is a research program that can move beyond whether any gene–activity interplay exists toward identifying the most intervention-relevant leverage points, while maintaining credibility through transparent design, reporting, and replication.

## 6. Conclusions

This review indicates that the most defensible, cross-design conclusion is not that a single genotype reliably determines who benefits from physical activity, but that physical activity is generally associated with lower depression risk and symptom burden even when genetic liability is explicitly modeled. Evidence from polygenic stratification studies suggests an additive pattern: higher depression polygenic risk elevates baseline risk, yet higher activity remains beneficial across PRS strata and can meaningfully offset inherited liability at the population level.

Across genetics-informed causal approaches, findings further support a protective role of physical activity—most consistently when activity is measured objectively (e.g., accelerometry) rather than via self-report, which is more vulnerable to misclassification and mood-related reporting bias. At the same time, the evidence base reinforces a dynamic, bidirectional framework: depression liability can also suppress physical activity, creating feedback loops that complicate interpretation of observational associations and highlight the importance of supporting initiation and maintenance of activity in those with emerging symptoms.

By contrast, candidate–gene moderation findings are mixed and context-dependent, tending to appear only under specific conditions (e.g., particular developmental windows, stress histories, symptom dimensions, or exposure operationalizations) rather than as stable, generalizable interaction effects. Methodologically, the field is constrained by chronic underpowering for interaction effects, heterogeneous depression phenotyping, fragmented physical-activity definitions, limited ancestral diversity (and therefore limited PRS portability), and the need for stronger triangulation across complementary designs.

Taken together, these findings support a pragmatic clinical and public-health implication: promoting physical activity remains justified broadly, including among individuals at higher genetic risk, but genotype-stratified “precision exercise prescriptions” are premature without replicated, well-powered effect modification and careful equity-focused validation. Future progress will depend on large harmonized multi-cohort efforts, improved and comparable activity measurement (preferably device-based with contextual enrichment), interaction-aware confounding control, diverse-ancestry validation, and deliberate triangulation combining cohorts, trials, within-family designs, and robust MR workflows.

## Figures and Tables

**Table 1:** Studies included in the review.

Study	Sample/Design	Genetic Factor	Physical Activity Exposure	Depression Outcome	Main Finding on Gene × Activity/Activity × Genetic Risk
[17]	Swedish primary care adults with mild–moderate depression; prospective cohort nested in RCT	BDNF Val66Met + childhood adversity	12-week physical exercise, ICBT, or TAU	MADRS response	Met carriers without childhood adversity responded best, especially in exercise; adversity abolished this advantage.
[18]	170 kinesiology undergraduates; cross-sectional	5-HTTLPR	Self-reported weekly activity	BDI symptoms	Physical activity moderated genotype effect: at low activity, s-allele carriers had more symptoms than ll; at high activity pattern differed unexpectedly.
[19]	Older sedentary adults in LIFE-P RCT	BDNF Val66Met, 5-HTTLPR, APOE ε4	12-month PA program vs. health education	CES-D total and subscales	No overall antidepressant effect, but men—especially BDNF Met carriers—showed greater somatic symptom improvement in PA arm.
[20]	3123 Spanish adults; cross-sectional community study	BDNF Val66Met	Self-reported activity, hours/week, intensity	DSM-IV major depression	No genotype main effect, but hours of activity interacted with genotype: more hours linked to lower depression particularly in Met carriers, especially women.
[21]	1072 midlife adults; cross-sectional	BDNF Val66Met	Paffenbarger activity score	CES-D symptoms/CES-D ≥ 16	No evidence that BDNF moderated the activity–depression association.
[22]	99 older adults in exercise RCT	APOE ε4, BDNF Val66Met	6-month moderate vs. high-intensity cycling vs. control	DASS-21 depression/anxiety/stress	BDNF did not moderate exercise effects; APOE ε4 moderated stress, not depression.
[23]	82 healthy adolescent girls; cross-sectional	BDNF Val66Met	Self-reported activity level	CDI-S	Higher activity related to fewer symptoms only in Met carriers; no relation in Val/Val girls.
[24]	639 Taiwanese adults ≥ 54; prospective cohort	APOE ε4	Baseline exercise frequency	CES-D at 6 years	Exercise predicted fewer later symptoms mainly in non-carriers; ε4 carriers did not show the same benefit.
[25]	17,934 Taiwanese adults; case-control/biobank linkage	MTHFR rs17367504	Regular exercise vs. none	Clinical MDD	Exercise modified genetic risk: GG genotype predicted MDD mainly in non-exercisers; regular exercise appeared protective in GG carriers.
[26]	129 college students with follow-up; randomized exercise trial	5-HTTLPR	5-week supervised cycling vs. control	BDI change	Exercise reduced symptoms overall; l-carriers improved more than ss participants.
[27]	7968 adults in biobank; prospective EHR study	Depression polygenic risk score (PRS)	Weekly recreational activity hours	Incident depression	Higher activity lowered incident depression across all PRS strata; no strong multiplicative interaction.
[28]	Two-sample MR using GWAS summary data	Genetic instruments for PA and MDD	Objective vs. self-reported PA	MDD	Objectively measured PA showed causal protection against depression; no evidence MDD causally reduced activity in robust form.
[29]	1196 Dutch adolescents; longitudinal cohort	Plasticity index incl. BDNF, 5-HTTLPR, DRD2/4, COMT, etc.	Repeated self-reported PA	Depressive symptoms	Small reciprocal PA ↔ depression effects, but no convincing moderation by plasticity genes or BDNF/5-HTTLPR individually.
[30]	Swedish adolescent cohort across 3 waves	BDNF rs6265 (Val66Met) + childhood stress	Leisure-time PA frequency	DSRS symptoms	Context-dependent moderation: PA moderated genotype effects in early adolescence; stress was stronger moderator in mid-adolescence, especially for AA carriers.
[31]	113 older endurance athletes vs. controls	BDNF Val66Met	Long-term endurance training	BDI/GDS	Exercise buffered genotype differences: among controls, Val/Val had more symptoms than T-carriers; among athletes this genotype gap disappeared.
[32]	1051 older adults with diabetes	BDNF Val66Met	PA index and grip strength	CES-D	PA related to fewer symptoms in both genotype groups; no significant PA × genotype interaction. Grip strength associations were more evident in Met carriers.
[33]	339,767 UK Biobank adults; prospective cohort	Depression PRS	Healthy lifestyle score including PA	Incident depression	Healthy lifestyle lowered risk across all genetic risk groups; no significant PRS × lifestyle interaction.
[34]	756 Danish twin pairs; twin modeling	Latent genetic liability	Fitness and exercise METs	Depression symptom composite	Higher fitness/activity linked to fewer symptoms and appeared to reduce variance in depression, including genetic variance for fitness.
[35]	76,218 UK Biobank adults with accelerometry	Depression PRS	Timing of daily activity/MVPA	Incident depression	Earlier activity timing associated with lower risk; PRS interaction not strong, though stratified patterns varied.
[36]	84,570 UK Biobank adults with accelerometry	Depression/anxiety PRS	Weekend-warrior vs. regular MVPA	Incident depression/anxiety	Meeting MVPA guidelines lowered risk regardless of pattern; benefit observed across genetic-risk strata.
[37]	UK Biobank + external GWAS; bidirectional MR	Genetic instruments for PA/ST and psychiatric traits	Accelerometer PA and sedentary time	Depression, anxiety, well-being	PA showed causal protection for depression and well-being; depression liability also appeared to causally reduce PA.
[38]	Two-sample bidirectional MR	Genetic instruments for PA/SB and psychiatric outcomes	Self-report and accelerometer PA/SB	Multiple psychiatric outcomes incl. MDD	Most robust psychiatric result: higher accelerometer-measured activity reduced depression risk.
[39]	Netherlands Twin Register; longitudinal twin/family study	Shared genetic liability	Leisure-time exercise	Anxiety/depression symptoms	Exercise-symptom association appeared largely due to shared genetic factors, not strong causal environmental effects.
[40]	Finnish Twin Cohort; co-twin control prospective study	Familial/genetic control via twin design	Persistent leisure-time PA across adulthood	Later antidepressant use	Persistently active twins had lower odds of later antidepressant use, suggesting association not fully explained by shared genes/family background.
[41]	Two-sample bidirectional MR	Genetic instruments for activity/inactivity and MDD	Leisure/social activity, physical activity, inactivity	MDD	Multiple activity types appeared protective, while physical inactivity increased MDD risk; reverse path MDD → inactivity also supported.
[42]	Two-sample MR	Genetic instruments for specific PA types	Walking, DIY, other exercise, strenuous sport, no PA	Depression/major depression	Walking for pleasure and heavy DIY showed protective causal estimates; no physical activity increased risk.
[43]	NHANES observational + MR	Genetic instruments for PA, sedentary time, sleep	PA, sedentary time, sleep duration	MDD/severe depressive symptoms	Observationally sedentary time and excessive sleep related to MDD; MR suggested aerobic/other exercise may causally reduce MDD risk.

**Table 2:** Risk of bias assessment (RoB-2).

Study	Bias Arising from the Randomization Process	Bias Due to Deviations from Intended Interventions	Bias Due to Missing Outcome Data	Bias in Measurement of the Outcome	Bias in Selection of the Reported Result
[17]	Low risk	Some concerns	High risk	Low risk	High risk
[19]	Some concerns	Some concerns	Some concerns	High risk	High risk
[22]	Some concerns	Some concerns	High risk	Some concerns	Some concerns to High risk
[26]	Some concerns	Some concerns	High risk	High risk	Some concerns

**Table 3:** Risk of bias assessment (ROBINS-I).

Study	Bias Due to Confounding	Bias in Selection of Participants into the Study	Bias in Classification of Interventions/Exposures	Bias Due to Deviations from Intended Exposures	Bias Due to Missing Data	Bias in Measurement of Outcomes	Bias in Selection of the Reported Result
[18]	Serious	Serious	Moderate	Low	Low to Moderate	Moderate	Moderate to Serious
[20]	Critical	Moderate to Serious	Serious	Low	Moderate	Low	Moderate to Serious
[21]	Serious	Moderate	Moderate	Low	Moderate	Moderate	Moderate
[23]	Serious	Moderate to serious	Moderate	Low	Moderate	Moderate	Moderate to serious
[24]	Serious	Moderate to Serious	Moderate	Moderate	Serious	Moderate	Moderate
[25]	Serious	Serious	Serious	Serious	Low to Moderate	Moderate	Serious
[27]	Serious	Moderate	Moderate	Low to Moderate	Moderate	Moderate	Moderate
[28]	Serious	Moderate	Low for accelerometer analysis; Serious for self-report analysis	Low	Moderate	Moderate	Moderate
[28]	Serious	Moderate to Serious	Serious	Low	Moderate	Moderate	Moderate
[30]	Serious	Serious	Moderate	Low	Serious	Moderate to Serious	Serious
[31]	Serious	Serious	Moderate	Low	Moderate	Moderate	Serious
[32]	Serious	Serious	Moderate	Low	Moderate to Serious	Moderate	Serious
[33]	Serious	Serious	Moderate	Moderate	Serious	Moderate	Moderate
[34]	Serious	Moderate to Serious	Moderate	Low	Moderate	Moderate	Serious
[35]	Serious	Moderate to Serious	Moderate	Low	Moderate	Moderate	Serious
[36]	Serious	Moderate	Low	Low	Low to Moderate	Moderate	Moderate
[37]	Serious	Serious	Low	Low	Moderate	Moderate	Moderate
[38]	Serious	Serious	Moderate	Low	Low to Moderate	Moderate	Moderate
[39]	Moderate to Serious	Moderate	Moderate	Low	Moderate to Serious	Moderate	Moderate
[40]	Serious	Moderate	Moderate	Low	Moderate	Low for the stated outcome; Moderate if interpreted as depression incidence	Moderate
[41]	Moderate	Moderate	Serious	Low	Low to Moderate	Moderate	Moderate to Serious
[42]	Serious	Moderate to Serious	Moderate	Low	Low to Moderate	Moderate	Serious
[43]	Serious	Serious	Serious	Low	Serious	Serious	Moderate

## Data Availability

No new data were created or analyzed in this study. Data sharing is not applicable to this article.

## Supplementary Materials

The following supporting information can be downloaded at: https://www.mdpi.com/article/10.3390/jcm15135025/s1. Reference [106] was cited in Supplementary Materials.
