# Supplementary material for: Gene–Physical Activity Interplay in Depression: Candidate–Gene Interactions, Polygenic Susceptibility, Lifestyle Context, and Mendelian Randomization Evidence—Systematic Review
Source: J Clin Med. 2026 Jun 27;15(13):5025. doi: 10.3390/jcm15135025 (PMC13362543; doi:10.3390/jcm15135025)
Supplement: Supplementary file 1 [file jcm-15-05025-s001.zip › jcm-4283259-Table S1.pdf]

Supplementary Table S1

Database / platform	Date searched	Search string	Filters / limits	Records retrieved
MEDLINE/PubMed	15 January 2026	(("depression"[Title/Abstract] OR "depressive symptoms"[Title/Abstract] OR "major depressive disorder"[Title/Abstract] OR "MDD"[Title/Abstract] OR "Depressive Disorder"[Mesh] OR "Depression"[Mesh] OR "CES-D"[Title/Abstract] OR "BDI"[Title/Abstract] OR "PHQ-9"[Title/Abstract] OR "MADRS"[Title/Abstract]) AND ("physical activity"[Title/Abstract] OR exercise[Title/Abstract] OR training[Title/Abstract] OR fitness[Title/Abstract] OR MVPA[Title/Abstract] OR sedentary[Title/Abstract] OR inactivity[Title/Abstract] OR accelerometer[Title/Abstract] OR walking[Title/Abstract] OR cycling[Title/Abstract] OR "Exercise"[Mesh] OR "Motor Activity"[Mesh] OR "Sedentary Behavior"[Mesh]) AND (genotype[Title/Abstract] OR polymorphism[Title/Abstract] OR "candidate gene"[Title/Abstract] OR BDNF[Title/Abstract] OR "5-HTTLPR"[Title/Abstract] OR APOE[Title/Abstract] OR MTHFR[Title/Abstract] OR SNP[Title/Abstract] OR "polygenic risk score"[Title/Abstract] OR PRS[Title/Abstract] OR GWAS[Title/Abstract] OR "Genome-Wide Association Study"[Mesh]) AND (interaction[Title/Abstract] OR moderation[Title/Abstract] OR "gene-environment"[Title/Abstract] OR "gene environment"[Title/Abstract] OR "GxE"[Title/Abstract] OR "G×E"[Title/Abstract] OR "Mendelian randomization"[Title/Abstract] OR "Mendelian randomisation"[Title/Abstract] OR	Humans; English; full-text peer-reviewed articles; no date restriction	11

		"instrumental variable"[Title/Abstract] OR "two-sample MR"[Title/Abstract]))		
Embase	15 January 2026	('depression'/exp OR 'major depression'/exp OR depression:ti,ab OR 'depressive symptoms':ti,ab OR 'major depressive disorder':ti,ab OR MDD:ti,ab OR 'CES-D':ti,ab OR BDI:ti,ab OR 'PHQ-9':ti,ab OR MADRS:ti,ab) AND ('physical activity'/exp OR 'exercise'/exp OR 'fitness'/exp OR 'sedentary lifestyle'/exp OR 'physical activity':ti,ab OR exercise:ti,ab OR training:ti,ab OR fitness:ti,ab OR MVPA:ti,ab OR sedentary:ti,ab OR inactivity:ti,ab OR accelerometer:ti,ab OR walking:ti,ab OR cycling:ti,ab) AND ('genotype'/exp OR 'genetic polymorphism'/exp OR 'genome-wide association study'/exp OR genotype:ti,ab OR polymorphism:ti,ab OR 'candidate gene':ti,ab OR BDNF:ti,ab OR '5-HTTLPR':ti,ab OR APOE:ti,ab OR MTHFR:ti,ab OR SNP:ti,ab OR 'polygenic risk score':ti,ab OR PRS:ti,ab OR GWAS:ti,ab) AND (interaction:ti,ab OR moderation:ti,ab OR 'gene-environment':ti,ab OR 'gene environment':ti,ab OR GxE:ti,ab OR 'Mendelian randomization':ti,ab OR 'Mendelian randomisation':ti,ab OR 'instrumental variable':ti,ab OR 'two-sample MR':ti,ab)	Humans; English; articles; no date restriction	9
PsycINFO	15 January 2026	(DE "Depression" OR DE "Major Depression" OR TI(depression OR "depressive symptoms" OR "major depressive disorder" OR MDD OR "CES-D" OR BDI OR "PHQ-9" OR MADRS) OR AB(depression OR "depressive symptoms" OR "major depressive disorder" OR MDD OR "CES-D" OR BDI OR "PHQ-9" OR MADRS)) AND (DE "Physical Activity" OR DE "Exercise" OR TI("physical activity" OR exercise OR training OR fitness OR MVPA OR sedentary OR inactivity OR accelerometer OR walking OR cycling) OR AB("physical activity" OR exercise	Humans; English; peer-reviewed journal articles; no date restriction	15

		OR training OR fitness OR MVPA OR sedentary OR inactivity OR accelerometer OR walking OR cycling)) AND (DE "Genetics" OR DE "Genetic Influences" OR TI(genotype OR polymorphism OR "candidate gene" OR BDNF OR "5-HTTLPR" OR APOE OR MTHFR OR SNP OR "polygenic risk score" OR PRS OR GWAS) OR AB(genotype OR polymorphism OR "candidate gene" OR BDNF OR "5-HTTLPR" OR APOE OR MTHFR OR SNP OR "polygenic risk score" OR PRS OR GWAS)) AND (TI(interaction OR moderation OR "gene-environment" OR "gene environment" OR GxE OR "Mendelian randomization" OR "Mendelian randomisation" OR "instrumental variable" OR "two-sample MR") OR AB(interaction OR moderation OR "gene-environment" OR "gene environment" OR GxE OR "Mendelian randomization" OR "Mendelian randomisation" OR "instrumental variable" OR "two-sample MR"))		
Web of Science Core Collection	15 January 2026	TS=(depression OR "depressive symptoms" OR "major depressive disorder" OR MDD OR "CES-D" OR BDI OR "PHQ-9" OR MADRS) AND TS=("physical activity" OR exercise OR training OR fitness OR MVPA OR sedentary OR inactivity OR accelerometer OR walking OR cycling) AND TS=(genotype OR polymorphism OR "candidate gene" OR BDNF OR "5-HTTLPR" OR APOE OR MTHFR OR SNP OR "polygenic risk score" OR PRS OR GWAS) AND TS=(interaction OR moderation OR "gene-environment" OR "gene environment" OR GxE OR "G×E" OR "Mendelian randomization" OR "Mendelian randomisation" OR "instrumental variable" OR "two-sample MR")	English; article or review screened for original studies; no date restriction	13
Scopus	15 January 2026	TITLE-ABS-KEY(depression OR "depressive symptoms" OR "major depressive disorder" OR MDD OR "CES-D" OR BDI OR "PHQ-9" OR MADRS) AND TITLE-ABS-KEY("physical activity" OR exercise OR training OR fitness OR MVPA OR sedentary OR inactivity OR accelerometer OR	English; article; no date restriction	12

		walking OR cycling) AND TITLE-ABS-KEY(genotype OR polymorphism OR "candidate gene" OR BDNF OR "5-HTTLPR" OR APOE OR MTHFR OR SNP OR "polygenic risk score" OR PRS OR GWAS) AND TITLE-ABS-KEY(interaction OR moderation OR "gene-environment" OR "gene environment" OR GxE OR "G×E" OR "Mendelian randomization" OR "Mendelian randomisation" OR "instrumental variable" OR "two-sample MR")		
Cochrane CENTRAL	15 January 2026	("depression" OR "depressive symptoms" OR "major depressive disorder" OR MDD OR "CES-D" OR BDI OR "PHQ-9" OR MADRS) AND ("physical activity" OR exercise OR training OR fitness OR MVPA OR sedentary OR inactivity OR accelerometer OR walking OR cycling) AND (genotype OR polymorphism OR "candidate gene" OR BDNF OR "5-HTTLPR" OR APOE OR MTHFR OR SNP OR "polygenic risk score" OR PRS OR GWAS) AND (interaction OR moderation OR "gene-environment" OR "gene environment" OR GxE OR "Mendelian randomization" OR "Mendelian randomisation" OR "instrumental variable" OR "two-sample MR")	Trials; English; no date restriction	10
